# Fungal response to drought in the maize rhizosphere after reusing cover crop root channels

**DOI:** 10.1093/ismeco/ycag273

**Published:** 2026-09-25

**Authors:** Debjyoti Ghosh, Yijie Shi, Iris M Zimmermann, Tobias Stürzebecher, Katja Holzhauser, Martin von Bergen, Anne-Kristin Kaster, Sandra Spielvogel, Michaela A Dippold, Jochen A Müller, Harald Kellner, Nico Jehmlich

**Affiliations:** Department of Molecular Toxicology, Helmholtz Centre for Environmental Research – UFZ GmbH, Permoserstraße 15, 04318 Leipzig, Saxony, Germany; Department of Applied Microbial Ecology, Helmholtz Centre for Environmental Research – UFZ GmbH, Permoserstraße 15, 04318 Leipzig, Saxony, Germany; Institute of Plant Nutrition and Soil Science, Department of Soil Science, Kiel University, Hermann-Rodewald-Straße 2, 24118 Kiel, Schleswig-Holstein, Germany; Institute of Plant Nutrition and Soil Science, Department of Soil Science, Kiel University, Hermann-Rodewald-Straße 2, 24118 Kiel, Schleswig-Holstein, Germany; Biogeochemistry of Agroecosystems, University of Göttingen, Büsgenweg 2, 37077 Göttingen, Lower Saxony, Germany; Institute of Crop Science and Plant Breeding, Agronomy and Crop Science, Kiel University, Ludewig-Meyen-Straße 9, 24118 Kiel, Schleswig-Holstein, Germany; Department of Molecular Toxicology, Helmholtz Centre for Environmental Research – UFZ GmbH, Permoserstraße 15, 04318 Leipzig, Saxony, Germany; Institute for Biochemistry, Faculty of Biosciences, Pharmacy and Psychology, University of Leipzig, Brüderstraße 34, 04103 Leipzig, Saxony, Germany; German Centre for Integrative Biodiversity Research (iDiv) Halle-Jena-Leipzig, Puschstraße 4, 04103 Leipzig, Saxony, Germany; Institute for Biological Interfaces (IBG 5), Karlsruhe Institute of Technology, Hermann-von-Helmholtz-Platz 1, 76344 Eggenstein-Leopoldshafen, Baden-Württemberg, Germany; Institute of Plant Nutrition and Soil Science, Department of Soil Science, Kiel University, Hermann-Rodewald-Straße 2, 24118 Kiel, Schleswig-Holstein, Germany; Geo-Biosphere Interactions, Department of Geosciences, University of Tübingen, Schnarrenbergstraße 94-96, 72076 Tübingen, Baden-Württemberg, Germany; Institute for Biological Interfaces (IBG 5), Karlsruhe Institute of Technology, Hermann-von-Helmholtz-Platz 1, 76344 Eggenstein-Leopoldshafen, Baden-Württemberg, Germany; Department of Bio- and Environmental Sciences, International Institute Zittau, Dresden Institute of Technology, Markt 23, 02763 Zittau, Saxony, Germany; Department of Molecular Toxicology, Helmholtz Centre for Environmental Research – UFZ GmbH, Permoserstraße 15, 04318 Leipzig, Saxony, Germany

**Keywords:** fungal community, drought, soil types, ITS2 sequencing, CAZyme, metaproteomics

## Abstract

Root channels formed by winter cover crop can enhance subsoil water and nutrient access for subsequent crops such as maize (*Zea mays* L.). Structure and function of fungal communities in these habitats remain poorly understood, especially under drought. We conducted a field experiment across three different soil types in northern Germany (Luvisol, Podzol, and Phaeozem) planted with different cover crop mixtures containing *Brassicaceae*, *Fabaceae*, and *Poaceae*, followed by maize cultivation under contrasting water regimes (drought vs. rainfall-fed). Drought-induced shifts in maize rhizosphere fungal communities and their functional responses within reused cover crop root channels were assessed using a multi-omics approach combining ITS2 amplicon sequencing, quantitative PCR and metaproteomics. Drought consistently restructured fungal communities, with relative abundances of *Ascomycota* and *Zoopagomycota* increasing and that of *Chytridiomycota* and *Mucoromycota* decreasing, alongside soil- and depth-specific shifts in fungal trophic modes, with pronounced subsoil responses. Metaproteomics revealed that drought responses were either via enhanced antioxidant defence mechanisms or by reduced levels of carbon and nitrogen metabolism-associated proteins, suggesting energy conservation strategies. Further, drought-stimulated upregulation of fungal cellulose- and hemicellulose-degrading and redox-active enzymes may have enhanced soluble sugar release and redox balancing, supporting osmotic homeostasis and metabolic flexibility under water limitation. These responses were strongly influenced by soil types, being most pronounced in the drought-prone Podzol and Luvisol and markedly buffered in the water-retentive Phaeozem. Together, our results demonstrate substantial structural and functional plasticity of rhizosphere fungal communities in reused root channels under water limitation, highlighting their potential role in microbiome-mediated drought resilience in agroecosystems.

## Graphical abstract

**Graphical abstract:**
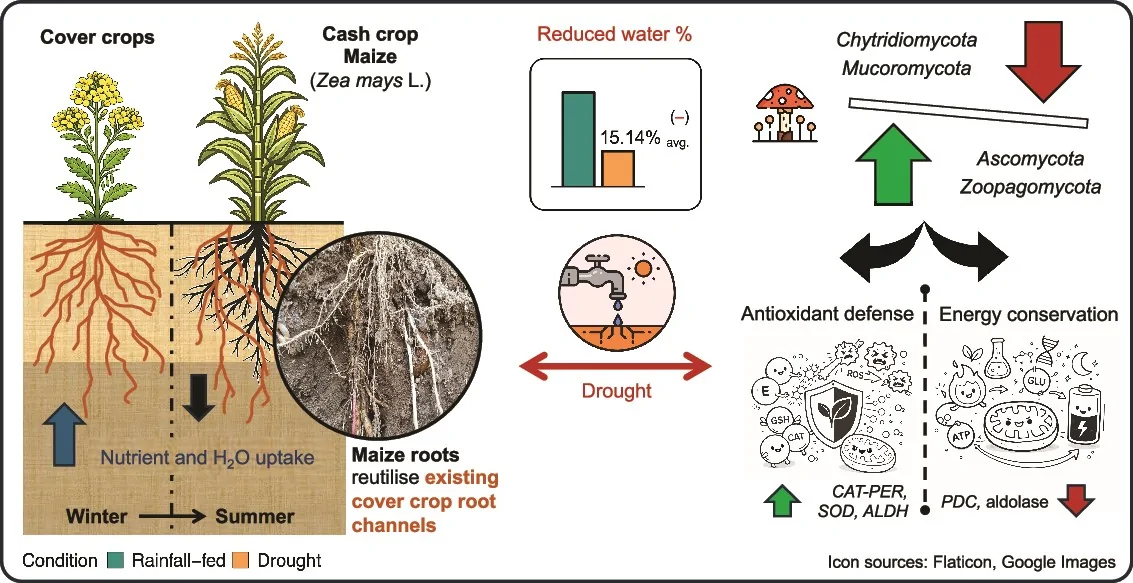

## Introduction

Drought is a major constraint on global crop productivity and disrupts biogeochemical processes in agricultural soils. Its frequency and severity have increased in recent decades [1, 2] driven by rising temperatures and long-term land use change [3]. As topsoil dries and nutrient mobility declines, plants may redirect root growth to deeper, less exposed subsoil layers. Enhancing the capacity of crops to exploit subsoil resources therefore represents a promising strategy to sustain productivity under water and nutrient limitation [4]. Root channels formed by preceding crops restructure soil microhabitats by modifying physical accessibility and microbial activity [5–7]. These stable macropores improve aeration, reduce mechanical resistance and facilitate deeper root penetration by subsequent crops. Beyond structural legacy, cover crops also enhance soil organic matter accumulation through residue decomposition, improving aggregate stability and water retention capacity. Cover crop mixtures, in particular, can improve soil hydraulic properties more effectively than monocultures owing to their complementary root architectures.

We previously showed that maize roots reusing winter cover crop root channels harbour higher bacterial abundance and activity in the rhizosphere [5], contributing to mitigation of drought-like conditions [8]. In particular, bacterial communities exhibited upregulation of reactive oxygen species (ROS)-detoxifying enzymes, including catalase, glutathione peroxidase and superoxide dismutase, under water limitation [8]. Whether fungal communities in these biopores display comparable adaptive responses remains unknown.

Fungi are key components of rhizosphere functioning, mediating soil structure, organic matter turnover, plant nutrient acquisition and stress tolerance properties [9–11], organic matter decomposition [12], and plant-fungi mutualistic relations. Their distribution is highly heterogenous and shaped by soil depths, physicochemical properties and management practices [13, 14]. Furthermore, soil type exerts strong control over fungal community composition [15]. Contrasting soil types can differ markedly in texture and chemistry, with Luvisols and Phaeozems characterized by high-activity clays and dark, loamy organic matter respectively, and both holding a high base status, whereas Podzols are sandy and acidic [16, 17]. Such differences influence the relative abundance of major fungal taxa and their vertical distribution along soil profiles [10].

Fungi are often considered comparatively drought-tolerant due to their filamentous growth form, which enables resource redistribution under low water availability [18, 19]. However, their functional responses to drought in agricultural soils remain poorly resolved. The ecological roles of mycorrhizal symbionts, saprotrophs and pathotrophs under stress depend on their capacity to mitigate oxidative damage and maintain metabolic homeostasis [20, 21]. While oxidative stress responses have been described in plant-associated and clinical fungi, soil fungal proteomic responses particularly within reused cover crop root channels remain largely unexplored. This knowledge gap limits our understanding of how cover cropping strategies shape fungi-mediated soil processes under climate-induced stress.

Here, we addressed this gap through field experiments in which maize followed winter cover crop mixtures of *Brassicaceae* (Brassica), *Fabaceae* (Legume), and *Poaceae* (Grass), with drought imposed using rainout shelters. Across soils of contrasting texture, we combined ITS2 amplicon sequencing, quantitative PCR (qPCR), and metaproteomics to investigate the effects of drought versus rainfall-fed cultivation on rhizosphere fungal communities of maize reusing pre-existing root channels. We hypothesized that fungal communities exhibit enhanced activity and distinct adaptive strategies under drought, modulated by soil type. By linking community composition with functional protein values across topsoil and subsoil, this study provides mechanistic insight to inform cover crop selection and strengthen crop resilience in water-limited agroecosystems.

## Materials and methods

### Crop cultivation and sampling regimes

Crops were grown in three agricultural fields – at experimental estates Hohenschulen of the Kiel University (Achterwehr, Germany, 54°18′44” N, 9°59′46″ E), Karkendamm of the Kiel University (Bad Bramstedt, Germany, 53°55′52” N, 9°55′15″ E), and Reinshof of the Georg-August-University of Göttingen (Rosdorf, Germany, 51°29′05” N, 9°53′34″ E), as described in a previous study [8]. The cash crop in this study was maize (*Zea mays* L.). Plots without cover crops during the winter (bare fallow) were established as control and compared against two cover crop mixtures on distinct plots. The cover crops were shallow- and deep-rooting *Brassicaceae* (*Brassica napus* L., rapeseed, shallow-rooting; *Raphanus sativus* L. var. *oleiformis*, oilseed radish, deep-rooting); *Fabaceae* (*Trifolium repens* L., white clover, shallow-rooting; *Trifolium pratense* L., red clover, deep-rooting); and *Poaceae* (*Lolium perenne*, perennial ryegrass, shallow-rooting; *Festuca arundinaceae*, tall fescue, deep-rooting), where the mixtures comprised of a combination of the shallow- and deep-rooting cover crops complementing the niche complementarity principle, reported to allow polycultures to overyield when plants compete for resources. All the cover crops were sown in October 2022 and grew until May 2023 in distinct randomized plots with four replicates of each variation, following a randomized complete block design pattern. In May 2023, a herbicide formulation (Roundup, Bayer AG, Leverkusen, Germany) was applied to all experimental plots (including fallow plots) to terminate all cover crops, and subsequently maize was sown in the same plots with the cover crop variations in addition to the fallow plots. Maize was grown in the fields from May to September 2023. To compare drought-like conditions against normal conditions, we artificially induced dry conditions using interrow rainout shelters and continued with the approach after observing differences in soil moisture trends between the sheltered and non-sheltered profiles using time-domain reflectometry sensors (Fig. S1a-c). A depiction of our rainout shelter setup used in the experiment has been provided within the figure of the experimental plan (Fig. 1). Further details about the results have been provided in the Extended data.

**Figure 1:**
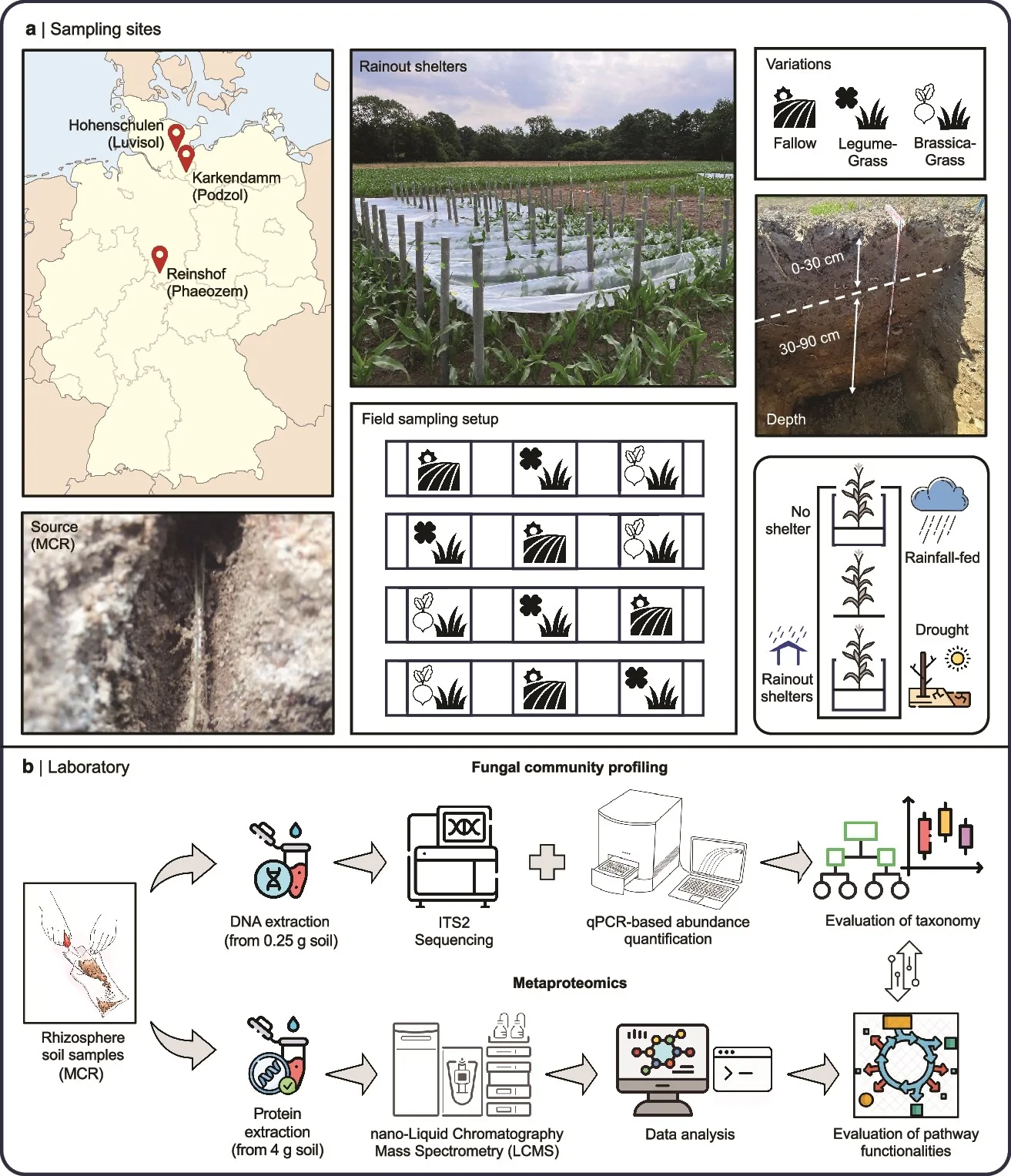
A schematic representation of the experimental design for studying reuse of winter cover crop root channels to cultivate maize. (a) Field sites: samples from Luvisol, Podzol, and Phaeozem in Germany with comparison of fungal microbiome associated with roots of maize (*Zea mays* L.) in root channels of cover crop [Legume/Grass (*Fabaceae*/*Poaceae*), Brassica/Grass (*Brassicaceae*/*Poaceae*)] or fallow (no cover crops) as control conditions. To replicate drought conditions, plots were covered with rainout shelters. (b) Community profiling using ITS2 gene-based amplicon sequencing, qPCR, and metaproteomics were used to visualize how the composition and functional role of fungal communities in the maize rhizosphere changed after being exposed to drought. Rhizosphere samples were taken from the maize root-reused cover crop root channels (MCR).

The sampling was done during the R1-RX growth stage of maize (bolting) grown in the Luvisol (01 August 2023), the Phaeozem (19 July 2023) and the Podzol (26 July 2023), and the profiles were maintained until sampling was completed around the flowering and reproduction stage of maize. No repetition of sampling was done from the profiles of the same plot in order to avoid bias and duplicates. To minimize potential operator bias when identifying “root overlap regions”, we used a randomized sampling scheme with the four blocks in Fig. 1 representing the four replicates and the ambient (natural rainfall) and drought (with interrow rainout shelters) plots always paired in direct proximity to each other.

### Soil physicochemical properties

Soil samples were oven-dried at 105°C for 24 h and milled. 10 mg of soil samples were weighed into tin capsules for analysis of total organic carbon (TOC) and nitrogen (TN) content using an elemental analyzer (ECS 4010, Costech Analytical Technologies, Valencia, USA). Soil cylinder samples were taken to measure soil bulk density at each soil depth. Soil moisture content was also measured by water content sensors (Teros 10, Meter Group, München, Germany), which were installed in the control plots (in 0–30 cm, 30–60 cm, and 60–90 cm depth under rainout shelter and on the rainfall-fed side) at each experimental site.

To investigate the influence of soil physicochemical properties on the fungal microbiome in the maize root channels, we correlated the parameters pH, soil moisture content, TOC, TN, and soil bulk density with the ITS2 gene amplicon data using Partial Mantel’s test [22] using the R package *microeco* (v1.10) [23]. The soil physicochemical properties were correlated to individual fungal phyla along the different soil types and soil moisture conditions to understand the impacts of these factors on the individual communities.

### ITS2 gene amplicon sequencing

Fungal community composition in the root-vicinity samples was analysed by sequencing amplicons targeting the fungal internal transcribed spacer (ITS) regions of rRNA (2^*^300 bp) on an Illumina NextSeq™ 550 (Illumina, San Diego, CA, USA). DNA was extracted from 0.25 g of soil using the DNeasy® PowerSoil® Pro Kit (QIAGEN GmbH, Hilden, Germany). PCR amplicons of the ITS2 region of the fungal rRNA gene were prepared using the forward and reverse primers fITS7 and ITS4 and the NEBNext®Ultra™ II Q5® Master Mix (New England Biolabs GmbH, Frankfurt, Germany) [24]. Sequencing libraries were prepared from 100 ng of DNA according to the Illumina protocol. Dual index adapters for the sequencing were attached using the NEBNext Multiplex Oligos for Illumina. The final concentration of the libraries was 2 nM after pooling. We sequenced triplicates of samples from each soil depth and root-vicinity source per cover crop variation plot for all three sampling sites (ITS2 gene amplicons, *n* = 147).

The sequencing data were analysed using QIIME2 v2023.5 [25]. First, the raw sequence reads were demultiplexed and quality-filtered (q-score 25) using the q2-demux plugin, followed by denoising with q2-dada2. The ITS2 regions of the fungal rRNA were trimmed to 230 bp for both the forward and reverse sequences. All amplicon sequence variants (ASVs) were aligned with q2-alignment, and then maximum-likelihood trees were constructed using q2-phylogeny. We chose ASV-based methods over OTU approaches to limit the effect of spurious taxa on diversity indices [26]. Taxonomic assignment of fungal ASVs was carried out using the q2-feature-classifier and the classify-sklearn Naïve Bayes taxonomy classifier against the UNITE v10.0 database [27] for QIIME2 (released on 04.04.2024). ASVs with a relative abundance of <0.01% were defined as rare taxa.

### Quantitative PCR

The copy number of the fungal ITS2 gene per gram of soil was quantified by SYBR® Green-based qPCR using a 7500 Fast Real-Time PCR System (Applied Biosystems™, Thermo Fisher Scientific, Waltham, MA, USA). Aliquots of the same DNA extract utilized in amplicon sequencing were used for qPCR. Dilutions of template DNA were used to compensate for the effect of PCR inhibitors in the samples. Each sample was analysed in triplicate. A PCR amplicon of the ITS2 region derived from *Trametes versicolor* (DSM 11269) was used to generate the standard curve. Each 20 μL reaction contained 1 μL of template DNA, the forward and reverse primers fITS7 and ITS4 for the ITS2 gene [24] without adapter nucleotides and Luna® Universal qPCR Master Mix (NEB). Reaction conditions were an initial denaturation for 1 min at 95°C, followed by 40 cycles of denaturation at 95°C for 15 s and extension at 60°C for 30 s. The melting curve was recorded in the temperature range of 60°C to 95°C. The gene copy numbers per gram of soil were determined in comparison against the standard essentially as before [28]. The average efficiency value was 100.8% ± 3.2%. The absolute ITS2 gene copies are provided in Table 1.

**Table 1:** Summary of ITS2 gene copies per gram soil for different parameters in our study. We quantified the mean and SD values of ITS2 gene copies per gram soil in soils from the reused cover crop root channels for all study parameters. The significance of each category is represented using compact letter display (CLD) calculated after conducting MANOVA on the gene copies under categories of soil type, cover crop variations and soil sampling depths. The values are provided in the Supplemental Tables S3a, S3e. ITS2 gene copies per gram of soil for individual fungal phyla has been provided in the Supplemental Table S3d.

Variation	Soil type	Depth	Condition	ITS2 gene copies g soil^−1^ (mean + SD)	CLD
Brassica/Grass	Luvisol	Topsoil	Rainfall-fed	3.09E+06 ± 2.33E+05	ab
			Drought	1.25E+07 ± 1.49E+06	
		Subsoil	Rainfall-fed	7.46E+06 ± 1.96E+06	abc
			Drought	1.03E+06 ± 1.21E+05	
	Phaeozem	Topsoil	Rainfall-fed	3.68E+07 ± 4.63E+06	a
			Drought	3.17E+07 ± 9.51E+06	
		Subsoil	Rainfall-fed	1.39E+05 ± 1.58E+04	cd
			Drought	6.64E+05 ± 7.72E+04	
	Podzol	Topsoil	Rainfall-fed	5.92E+05 ± 6.32E+04	bc
			Drought	1.29E+07 ± 3.62E+06	
		Subsoil	Rainfall-fed	1.35E+05 ± 1.82E+04	cd
			Drought	6.86E+05 ± 9.41E+04	
Fallow	Luvisol	Topsoil	Rainfall-fed	9.36E+06 ± 1.94E+06	ab
			Drought	1.83E+07 ± 1.39E+06	
		Subsoil	Rainfall-fed	3.27E+06 ± 6.78E+05	abc
			Drought	8.91E+05 ± 9.49E+04	
	Phaeozem	Topsoil	Rainfall-fed	3.51E+07 ± 4.06E+06	ab
			Drought	8.54E+06 ± 1.52E+06	
		Subsoil	Rainfall-fed	8.87E+04 ± 1.61E+04	d
			Drought	2.35E+05 ± 3.37E+04	
	Podzol	Topsoil	Rainfall-fed	1.01E+07 ± 3.05E+06	ab
			Drought	3.51E+06 ± 5.36E+05	
		Subsoil	Rainfall-fed	2.40E+06 ± 2.50E+05	abc
			Drought	2.22E+06 ± 4.68E+05	
Legume/Grass	Luvisol	Topsoil	Rainfall-fed	2.68E+07 ± 3.45E+06	ab
			Drought	4.94E+06 ± 6.83E+05	
		Subsoil	Rainfall-fed	1.72E+06 ± 1.87E+05	bc
			Drought	8.41E+05 ± 1.01E+05	
	Phaeozem	Topsoil	Rainfall-fed	4.30E+07 ± 1.40E+07	ab
			Drought	1.65E+07 ± 1.65E+06	
		Subsoil	Rainfall-fed	6.05E+04 ± 5.25E+03	d
			Drought	2.80E+05 ± 5.70E+04	
	Podzol	Topsoil	Rainfall-fed	7.59E+06 ± 2.01E+06	ab
			Drought	6.74E+06 ± 1.51E+06	
		Subsoil	Rainfall-fed	1.90E+05 ± 2.01E+04	cd
			Drought	1.69E+05 ± 3.07E+04	

### Metaproteomics analysis

At each timepoint, samples were collected separately from three plots for each cover crop variation at the analysed soil depths and root-vicinity (rhizosphere) sources and used for proteomic analyses following previously described protocols [5, 8] (*n* = 119). Approximately 4 g of soil was used for protein extraction using the SDS buffered-phenol extraction method as previously described [5]. To estimate the proportion of extracellular carbohydrate-active enzymes (CAZymes), signal peptides were predicted using Phobius [29] and proteins harbouring a signal peptide were classified as extracellular.

Taxonomic identities were retrieved using ENTREZ identifiers from the NCBI database [30, 31] employing KEGG Orthology (KO) numbers as unique protein identifiers. Mapping identified proteins to NCBI entries enabled comprehensive taxonomic annotation of their source fungi. Integration of KO-based pathway annotations with taxonomic assignments allowed generation of datasets featuring both community composition and functional potential. Following pathway categorisation, proteins mapped to biogeochemical cycles of interest helped evaluate shifts associated with soil properties, moisture status, and the re-use of cover crop root biopores, thereby elucidating microbial functional responses.

To assess the impact of environmental stress following root channel re-use, fold changes in label-free quantification (LFQ) intensities under drought conditions were calculated relative to rainfall-fed controls. Proteins were considered differentially abundant when |log₂ fold change| >0.6 and *P* < .05 [32]. In addition, to quantify the effects of cover crop root channel re-use, differential abundances were determined by subtracting protein intensities measured under cover crop treatments (*Brassicaceae/Poaceae* and *Fabaceae/Poaceae* rotations) from those observed under fallow conditions. Further details about our metaproteomics approach have been provided in the Extended data.

### Statistical data analysis

We used R (v4.3.1) [33] to perform all statistical analyses of the sequencing and the metaproteomics data. All measures of significance were calculated using permutational multivariate analysis of variance (PERMANOVA), followed by adonis2 function from *vegan* (v2.6–4) for partitioning sum of squares using dissimilarities to denote significances [34]. In the ITS2 gene sequencing analysis, the ASV abundance tables were filtered with total-frequency-based filtering based on 95% sequence identity and rarefied at 30 000 sequences to ensure equal sampling depth and sorting in the maximum number of samples for diversity analyses. Alpha and beta diversity metrics were calculated using the packages *phyloseq* [35] and *metacoder* from R (v4.3.1) [33]. Observed ASV richness measured for each cover crop variation at different sampling sites and conditions was used for estimating alpha diversity richness. Pielou’s evenness is the most widely used diversity evenness index in the ecological literature [36]. For beta diversity, we used Bray–Curtis dissimilarity [37] and visualized differences via Principal Coordinate Analysis (PCoA) using *vegan* (v2.6–4). Significance of fungal absolute abundances between the different parameters was represented using Compact Letter Display (CLD) [38] after Log_10_-transformation of absolute gene copies to stabilize variance and analysed with univariate linear models including soil type, cover crop variations, depth and moisture conditions, followed by estimated marginal means (*emmeans* package) [39] averaged over moisture conditions and compared by Šidák-adjusted pairwise contrasts and summarized as CLD [40]. To account for the non-independence of taxa, Shannon and Simpson indices were additionally estimated using DivNet [41], which models taxon co-occurrence through a log-ratio formulation to the non-rarefied counts, and the effects of cover crop variations, soil type, depth and moisture conditions were tested jointly. For accounting the compositional nature of sequencing data, community composition was additionally analysed using a centred log-ratio transformation [42], with beta diversity quantified as the Aitchison distance and the same PERMANOVA model applied (adonis2). This analysis was performed on non-rarefied counts, as the log-ratio transformation accounts for differences in sequencing depth and rarefaction is therefore not required [43]. Homogeneity of multivariate dispersion was tested for soil type and depth using PERMDISP to identify the presence of dispersion effects [44]. For metaproteomics datasets, differences in protein expression among cover crop variations were evaluated using a multi-factor ANOVA (cover crop variation as the fixed effect; soil type, depth and moisture condition as additional factors), followed by Tukey’s HSD [45], for pairwise comparisons. Significance is indicated by stars based on adjusted p-values (^*^P < .05, ^**^P < .01, ^***^P < .001, ^****^P < .0001). All figures were generated in RStudio. Other integrated packages used for statistical analyses and figure generation were *tidyverse* (v2.0.0) [46], and *dplyr* (v1.1.3) [47].

## Results

### Fungal community structure in different soil types under drought stress

The study encompassed three soil types: Luvisol, Phaeozem, and Podzol, and two soil depths (topsoil: 0–30 cm; subsoil: 30–90 cm) (Fig. 1). Soil physicochemical properties differed significantly among soil types and between depths (Fig. S2, Table S1).

We obtained 37 437 unique ASVs assigned to 83 classes within 12 fungal phyla (Fig. S3). Fungal richness increased under drought relative to rainfall-fed when *Brassicaceae/Poaceae* was the cover crop mixture in all the soil types (Luvisol: 832_Drought_ vs 729_Rainfall-fed_, Phaeozem: 742_Drought_ vs 614_Rainfall-fed_, Podzol: 804_Drought_ vs 696_Rainfall-fed_). Using *Fabaceae/Poaceae*, richness increased significantly in the Luvisol and Podzol but declined in the Phaeozem (Luvisol: 878_Drought_ vs 729_Rainfall-fed_, Phaeozem: 511_Drought_ vs 579_Rainfall-fed_, Podzol: 873_Drought_ vs 800_Rainfall-fed_). The richness under fallow conditions declined in the Phaeozem compared with the Luvisol and Podzol, although no clear differences were noticeable between drought and rainfall-fed (Luvisol: 831_Drought_ vs 815_Rainfall-fed_, Phaeozem: 599_Drought_ vs 667_Rainfall-fed_, Podzol: 818_Drought_ vs 827_Rainfall-fed_) (Fig. 2a, Table S2a). Evenness increased only for *Fabaceae/Poaceae* (0.774*_Fabaceae/Poaceae_* > 0.766_Fallow_ > 0.764*_Brassicaceae/Poaceae_*), while no other changes were detected (Fig. 2a, Table S2b). These patterns were confirmed by checking non-independence among taxa after adjustment for all factors: both Shannon and Simpson diversity were dominated by depth (higher in the topsoil) and were higher under drought than under rainfall-fed conditions (Table S2c). Beta-diversity estimations using Bray–Curtis dissimilarity and Aitchison metrics revealed differences among Podzol fungal communities from those in the Luvisol and Phaeozem, as well as between the sampling depths (Fig. 2b, Table S2d, e), consistent with previously reported bacterial community behaviours in the same experimental system [8].

**Figure 2:**
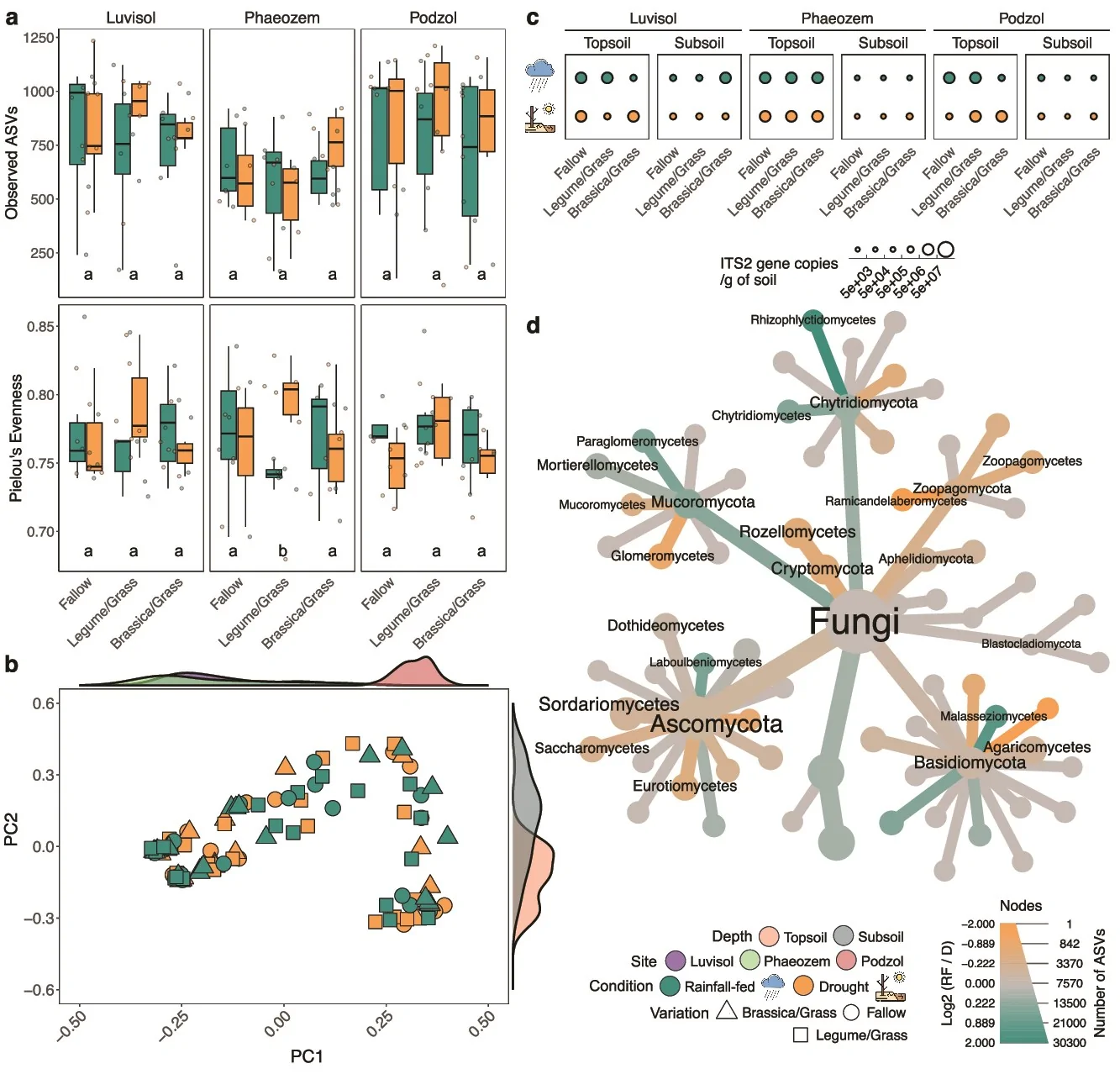
Graphical representation of the taxonomic diversity and absolute abundances at the different experimental sites under soil moisture conditions. (a) Alpha diversity richness (Observed ASVs) and evenness (Pielou’s evenness index) metrices reflecting the distribution of fungal communities at the three sampling sites under drought (D) and rainfall-fed (RF) conditions. Pairwise correlation between the variations is shown using a compact letter display representation for the significant differences between the variations, calculated using estimated margined means (with Šidák-adjusted pairwise contrasts); (b) Fungal community beta-diversity visualised using PCoA ordination based on Bray-Curtis dissimilarities along the sampling sites and depths for each cover crop variation under soil moisture conditions. Distance metrics were calculated from the identified ASVs. The different colours of the density plots next to the axes represent the soil types and sampling depths, and peak height and shape abundance; (c) ITS2 gene copies measured using qPCR as surrogate for absolute fungal abundances in the soil types, of each cover crop variation [Fallow, Legume/Grass (*Fabaceae*/*Poaceae*) and Brassica/Grass (*Brassicaceae*/*Poaceae*)] and soil moisture conditions (drought and rainfall-fed); (d) Taxonomic heat trees represent the differential abundance of the fungal phylotypes between two parameters of soil moisture content [drought (D) vs rainfall-fed (RF)]. The yellow-ochre colour indicates increases under drought conditions and green colour under rainfall-fed conditions. For Luvisol, *n* = 35; for Phaeozem, *n* = 36; and for Podzol, *n* = 35; for soil moisture conditions (drought and rainfall-fed) and depth (topsoil and subsoil), *n* = 106 (statistical details provided in Supplemental Table S2e–j; heat-tree fold-change data provided in Supplemental Table S3c).

Quantitative PCR analysis demonstrated a 12.8-fold reduction in ITS2 gene copy numbers from the topsoil to the subsoil (1.60 × 10^7^_Topsoil_ > 1.25 × 10^6^_Subsoil_) (Fig. 2c, Table 1). Absolute abundances were highest in the Phaeozem and declined sequentially in the Podzol and the Luvisol (1.44 × 10^7^_Phaeozem_ > 0.75 × 10^7^_Luvisol_ > 0.40 × 10^7^_Podzol_). Among cover crop treatments, *Fabaceae/Poaceae* resulted in the highest ITS2 gene copies, exceeding *Brassicaceae/Poaceae* and fallow (9.52 × 10^6^*_Fabaceae/Poaceae_* > 8.97 × 10^6^*_Brassicaceae/Poaceae_* > 7.83 × 10^6^_Fallow_). Under drought, fungal abundance increased within *Brassicaceae/Poaceae* root channels in the Luvisol and Podzol, but declined with *Fabaceae/Poaceae*. In the subsoil, *Fabaceae/Poaceae* had lower ITS2 gene abundances than *Brassicaceae/Poaceae* and fallow (subsoil: 1.69 × 10^6^*_Brassicaceae/Poaceae_* ≈ 1.52 × 10^6^_Fallow_ > 0.54 × 10^6^*_Fabaceae/Poaceae_*), although these differences were not statistically significant, as in the topsoil (Table 1, Table S3a).

Although overall richness and evenness limited cover crop mixture effects, taxon-specific responses were evident at the phylum and class levels (Table S4). The *Dikarya* subkingdom, comprising *Ascomycota* and *Basidiomycota*, showed minimal change under drought relative to rainfall-fed (*Log_2_FC:* 0.034 and 0.014, respectively) (Fig. 2d, Table S3b–c). However, within these phyla, individual classes responded divergently: *Laboulbeniomycetes* (−0.218) of *Ascomycota* and *Malasseziomycetes* (−0.531) of *Basidiomycota* decreased under drought. *Mucoromycota* and *Zoopagomycota* exhibited contrasting outcomes, with the former taxa declining under drought against rainfall-fed (−0.136) while the latter increasing (0.130). Within *Mucoromycota*, *Glomeromycetes* (arbuscular mycorrhizal fungi; 0.399) and *Mucoromycetes* (0.152), increased under drought and were more abundant in the Luvisol and Phaeozem. Similarly, classes within *Zoopagomycota* were enriched in these two soils but declined upon reusing cover crop root channels (Fig. S4). *Chytridiomycota* (−0.061) and *Cryptomycota* (0.254), displayed opposing abundance trends under drought and in the drought-prone Podzol.

Across soil depths, *Dikarya* exhibited vertical niche differentiation, with *Ascomycota* predominating in the topsoil and *Basidiomycota* in the subsoil. Exceptions included *Saccharomycetes* (*Ascomycota*) and the basidiomycete classes *Atractiellomycetes* and *Cystobasidiomycetes* (Fig. S5). Similar depth stratification was observed for *Mucoromycota* (enriched in subsoil) and *Zoopagomycota* (enriched in topsoil). The flagellated *Chytridiomycota* and the chitin cell-wall-lacking *Cryptomycota* preferentially inhabit the comparatively less compact topsoil. Although phylum-level composition showed limited responsiveness to cover crop treatments (Fig. S6), alpha diversity was significantly enriched under cover cropping – most strongly with *Brassicaceae/Poaceae* relative to fallow (Table S2c).

### Correlation of fungal communities with soil physicochemical properties

Soil physicochemical characteristics were examined previously, which showed that organic carbon content varied substantially with soil type and depth [8] (Fig. S2, Table S1). Partial Mantel tests revealed significant associations between fungal community composition and soil physicochemical properties (Fig. 3a, Table S5a–d). In the silt-rich Phaeozem, *Ascomycota* abundance correlated positively with TN and TOC in *Fabaceae/Poaceae* under both drought and rainfall-fed conditions. Associations with pH depended on the soil, ranging from negative in the Phaeozem to positive in the topsoil, where *Ascomycota* also correlated positively with soil moisture. *Basidiomycota* association trends were exactly opposite to *Ascomycota*—negatively correlating with TN and TOC, and with moisture in the topsoil. *Zoopagomycota* correlated positively with moisture and pH in the topsoil, whereas negative associations were observed in the alkaline Phaeozem. The other zygosporangia-forming lineage, *Mucoromycota,* showcased the reverse pattern. Also, chitin-lacking *Cryptomycota* and the rare *Aphelidiomycota* were negatively correlated with both moisture and pH.

**Figure 3:**
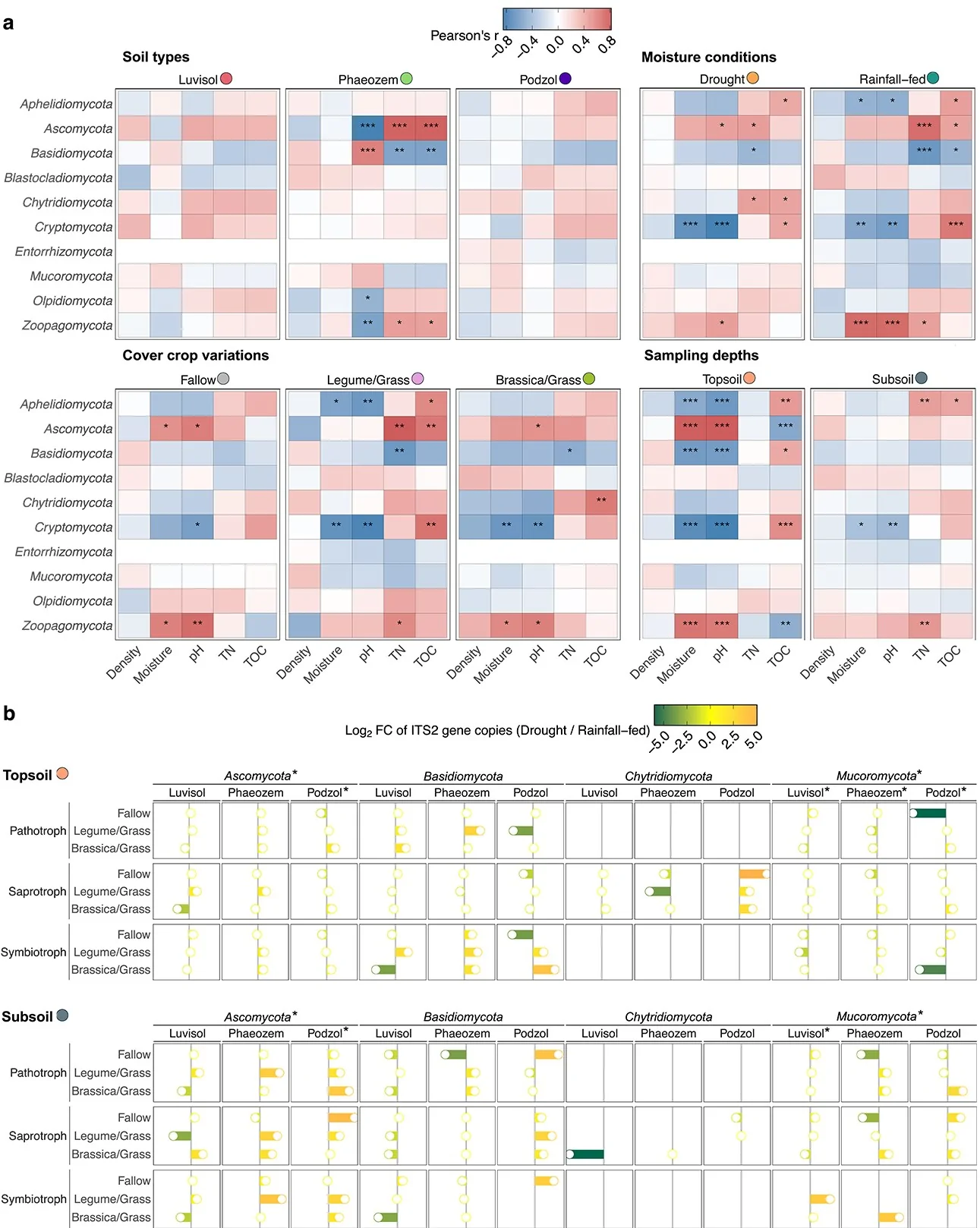
Graphs representing the correlation of individual fungal communities and the soil physicochemical properties across the parameters of study and the comparative abundance of fungal communities based on trophic modes. (a) The correlations between each fungal phylum with the physicochemical properties (TN, TOC, pH, moisture, and density) have been estimated for each cover crop variation, soil moisture conditions, soil types and sampling depths using partial Mantel’s test. Pearson’s correlation values and the significance estimation for each physicochemical protein and each phylum are provided in the Supplemental Table S5a–d; (b) The comparative abundance of the fungal communities classified by trophic modes, as defined in the FUNGuild database. This was estimated by *log_2_-FC* values of ITS2 gene copies per gram soil for drought versus rainfall-fed conditions. The yellow-ochre colour indicates increases under drought conditions and green colour under rainfall-fed conditions. The significant correlations are represented by asterisks inside the heatmap tiles; ^*^P < .05, ^**^P < .01, ^***^P < .001; Legume/Grass: *Fabaceae*/*Poaceae*, Brassica/Grass: *Brassicaceae*/*Poaceae*.

To link taxonomic observations with ecological attributes, fungal communities were mapped to the FUNGuild database [48] to trophic modes and compare the changes in abundances under drought versus rainfall-fed (Fig. 3b). Notable shifts along the trophic modes were visible in the subsoil as compared to the topsoil (Table S6a). The most significant changes involved *Mucoromycota*, which includes arbuscular mycorrhizal fungi (*Glomeromycetes*), in the topsoil of all three soil types and in the subsoil of Luvisol (Table S6b). For *Ascomycota*, changes were limited to the Podzol. With the effect of drought, topsoil pathotrophs from *Ascomycota* (plant pathotrophic *Eurotiomycetes* and *Sordariomycetes*), *Basidiomycota* (animal pathotrophic *Malasseziomycetes*) and *Mucoromycota* (animal pathotrophic *Mucoromycetes*) declined in the Podzol for *Fabaceae/Poaceae* and fallow but increased slightly for *Brassicaceae/Poaceae*. In contrast, subsoil pathotrophs increased with both cover crop treatments relative to bulk soil, particularly in the Podzol. Overall, saprotrophs and symbiotrophs generally increased following cover crop applications in the Phaeozem and Podzol. Notable exceptions were seen in the Luvisol, where *Basidiomycota* (*Agaricomycetes*) and *Chytridiomycota* (*Chytridiomycetes*) declined in *Brassicaceae/Poaceae* root channels, and in the Podzol topsoil, where for *Basidiomycota* and *Mucoromycota* (*Mucoromycetes*) symbiotrophs decreased significantly. The information about fungal trophic modes is provided in the supplemental tables S7a, S8a and S8c.

### Soil fungal metaproteome insights under drought

To assess the effects of drought on fungal biochemical pathways within reused cover crop root channels, proteins were identified by metaproteomics and assigned to taxonomic groups. Expression of fungal proteins varied significantly with soil type, depth, and cover crop mixture under drought stress (Table S9a). Protein groups trends were consistent with the ITS2 sequencing results, with some exceptions. Under drought, protein groups increased in the topsoil of the Luvisol and Podzol for *Brassicaceae/Poaceae* compared with RF (416_Luvisol-Drought_ > 356_Luvisol-Rainfall-fed_; 504_Podzol-Drought_ > 362_Podzol-Rainfall-fed_), but increased only in the Podzol subsoil and not in the Luvisol (284_Luvisol-Drought_ < 337_Luvisol-Rainfall-fed_; 336_Podzol-Drought_ > 294_Podzol-Rainfall-fed_) (Table S9b). For *Fabaceae/Poaceae*, protein groups increased in the Luvisol (429_Luvisol-Drought-Topsoil_ > 353_Luvisol-Rainfall-fed-Topsoil_; 341_Luvisol-Drought-Subsoil_ > 179_Luvisol-RF-Subsoil_) but showed no substantial change in Podzol topsoil, while increasing in Podzol subsoil (470_Podzol-Drought-Topsoil_ < 488_Podzol-Rainfall-fed-Topsoil_; 288_Podzol-Drought-Subsoil_ > 226_Podzol-Rainfall-fed-Subsoil_). In contrast, no clear shifts were seen in the Phaeozem for either cover crop mixture or under fallow conditions (364_*Fabaceae/Poaceae*-Drought-Topsoil_ and 361_*Fabaceae/Poaceae*-RF-Topsoil_; 213_*Fabaceae/Poaceae*-Drought-Subsoil_ and 229_*Fabaceae/Poaceae*-Rainfall-fed-Subsoil_; (365_*Brassicaceae/Poaceae*-Drought-Topsoil_ and 369_*Brassicaceae/Poaceae*-Rainfall-fed-Topsoil_; 219_*Brassicaceae/Poaceae*-__Drought-Subsoil_ and 211_*Brassicaceae/Poaceae*-Rainfall-fed-Subsoil_). Relative to rainfall-fed, drought altered protein expression in the moisture-limited soils, Luvisol and Podzol, following the use of cover crop mixtures. In the drought-prone sandy Podzol, overall protein expression increased only with *Brassicaceae/Poaceae* (*Log_2_FC:* 0.532), exceeding both fallow (0.017) and *Fabaceae*/*Poaceae* (−0.245) (Table S9c–e). Similarly, protein expressions in the Luvisol increased only with *Brassicaceae/Poaceae* (0.328_*Brassicaceae/Poaceae*-Log2FC_ > 0.076_Fallow-Log2FC_ > −0.470_*Fabaceae/Poaceae*-Log2FC_). By contrast, the Phaeozem had the highest water retention under drought and showed reduced protein expression following cover crop introduction relative to bulk soil (0.441_Fallow-Log2FC_ > 0.148_*Brassicaceae/Poaceae*-Log2FC_ > 0.044_*Fabaceae/Poaceae*-__Log2FC_).

Differential expression analysis revealed a greater number of upregulated proteins in the Luvisol and the Phaeozem (Fig. 4, Table S10). Fatty-acid-synthesising 3-hydroxydecanoyl-[acyl-carrier-protein (ACP)] dehydratase, and oxidative stress regulators aldehyde dehydrogenase (NAD^+^) (ALDH) and superoxide dismutase (SOD) were upregulated under drought (Table S7a). In contrast, enzymes of the pyruvate dehydrogenase complex (PDC) enzymes contributing to acetyl-CoA synthesis were downregulated, particularly in *Fabaceae/Poaceae* root channels. In the Luvisol, osmoprotectant glycerol-3-phosphate dehydrogenase (G3PDH) and glutamate racemase (MurI) of the N cycle were upregulated with *Brassicaceae/Poaceae* under drought. In the Podzol, membrane translocases and catalase peroxidase (CAT-PER) were upregulated, whereas phosphoribosylformylglycinamide synthase was downregulated. Methionine cycle enzymes, including homocysteine methyltransferase, methionine synthase (MeSe) and S-adenosylmethionine (SAM) synthase, were upregulated in *Fabaceae/Poaceae* root channels in the Luvisol.

**Figure 4:**
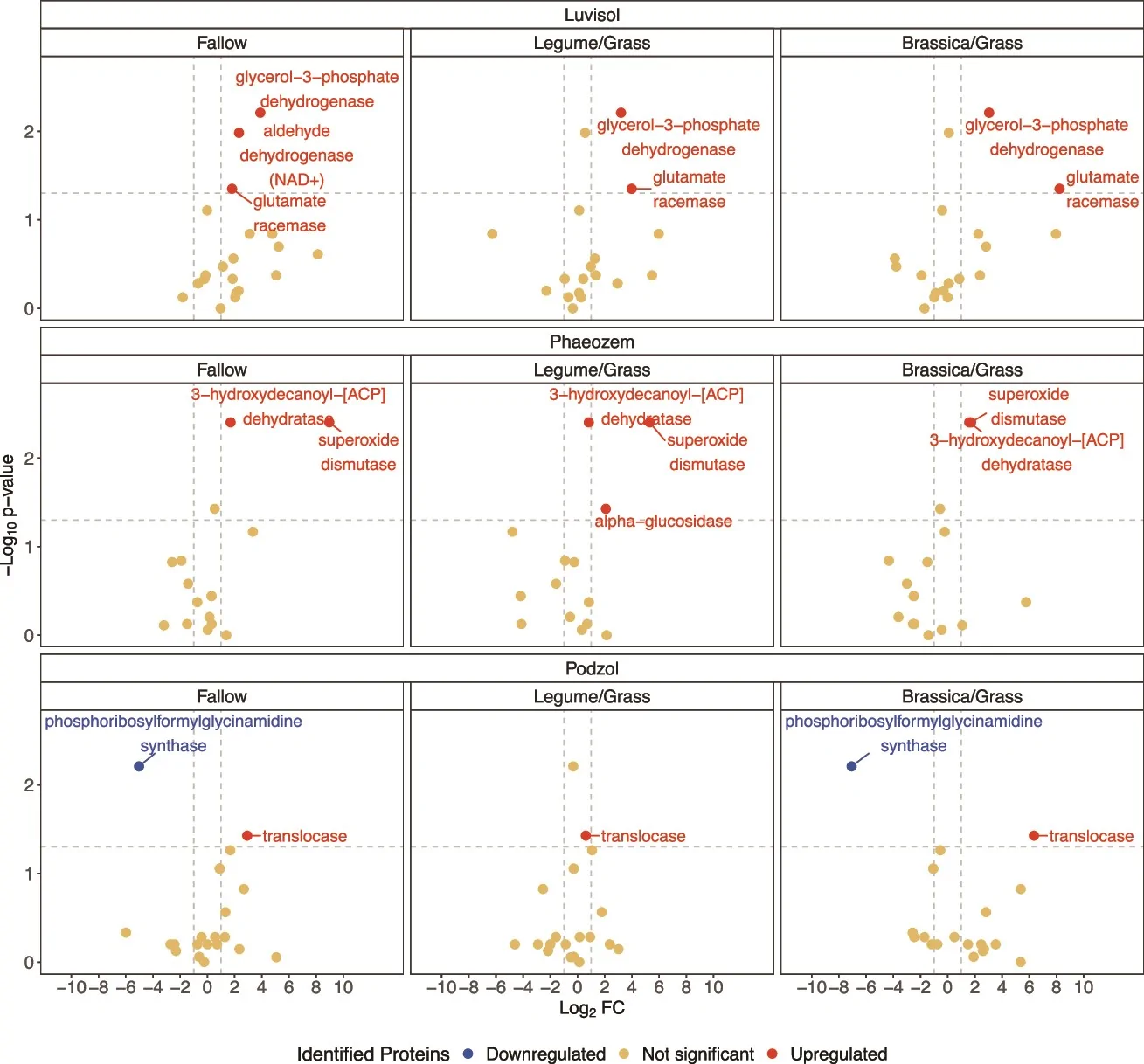
Depiction of upregulated and downregulated fungal proteins under drought. A volcano plot representation for the upregulation and downregulation of fungal proteins differing significantly under drought conditions at the three sampling sites (Luvisol, Phaeozem and Podzol) for the cover crop variations (Fallow, Legume/Grass and Brassica/Grass). The *x*-axis represents the l*og_2_ fold-change* (*FC*) and the *y*-axis represents the -*log_10_* p-value according to Mann–Whitney U test. The proteins having *log_2_FC* values <0.6 or > −0.6 and *P* > .05 were classified as “not significant”. l*og_2_FC* > 0.6 and *P* < .05 were the “upregulated” proteins and *log_2_FC* < 0.6 and *P* < .05 were the proteins “downregulated”. Names of several identified proteins are not shown to avoid crowdedness. The information for the all the represented significantly upregulated or downregulated proteins have been provided in the Supplemental Table S10; Legume/Grass: *Fabaceae*/*Poaceae*, Brassica/Grass: *Brassicaceae*/*Poaceae*.

Consistent with amplicon sequencing, *Ascomycota* produced the largest proportion of the identified proteins. Based on absolute LFQ intensities of highly expressed enzymes, 3-hydroxydecanoyl-[ACP]-dehydratase was expressed exclusively by *Ascomycota*, particularly in the Podzol. The same patterns were observed for MeSe and homocysteine methyltransferase, whereas aspartokinase and homoserine dehydrogenase and the oxidative stress regulator ALDH were more strongly expressed in the Luvisol (Fig. 5, Fig. S7, Table S7a). Beyond *Ascomycota*, SAM synthase expression from *Basidiomycota*, *Chytridiomycota*, *Mucoromycota* and *Zoopagomycota* was relatively higher in the Podzol, while G3PDH was expressed more in the Luvisol. ALDH expressions were similar from *Ascomycota* in both Luvisol and Podzol and from *Basidiomycota* in the Phaeozem. Additional stress responders showed soil-specific patterns: SOD was relatively upregulated in *Ascomycota* in the Phaeozem, while CAT-PER from *Basidiomycota* was overexpressed in the Podzol inside *Brassicaceae/Poaceae* root channels. The pentose phosphate pathway enzyme 6-phosphogluconolactonase (6-PGL) was more strongly expressed by *Ascomycota* in the Podzol under drought. Despite a relative abundance below 1%, *Blastocladiomycota* contributed substantially to glycolytic aldolase expression. The Podzol also represented a higher abundance of N cycle enzymes, glutamate dehydrogenase and urease, primarily contributed by *Mucoromycota*, *Basidiomycota* and *Chytridiomycota*. Glutamate racemase was expressed highly in the Luvisol under drought. In the topsoil, *Ascomycota*, *Basidiomycota* and *Mucoromycota* were the top taxa contributing the most to the proteome (Table S7b). However, in the subsoil, *Mucoromycota* exceeded those of *Basidiomycota*, while overall protein expressions from other phyla declined relative to topsoil. An extended heatmap of fungal protein expression across all the study parameters is provided in the supplementary fig. S8.

**Figure 5:**
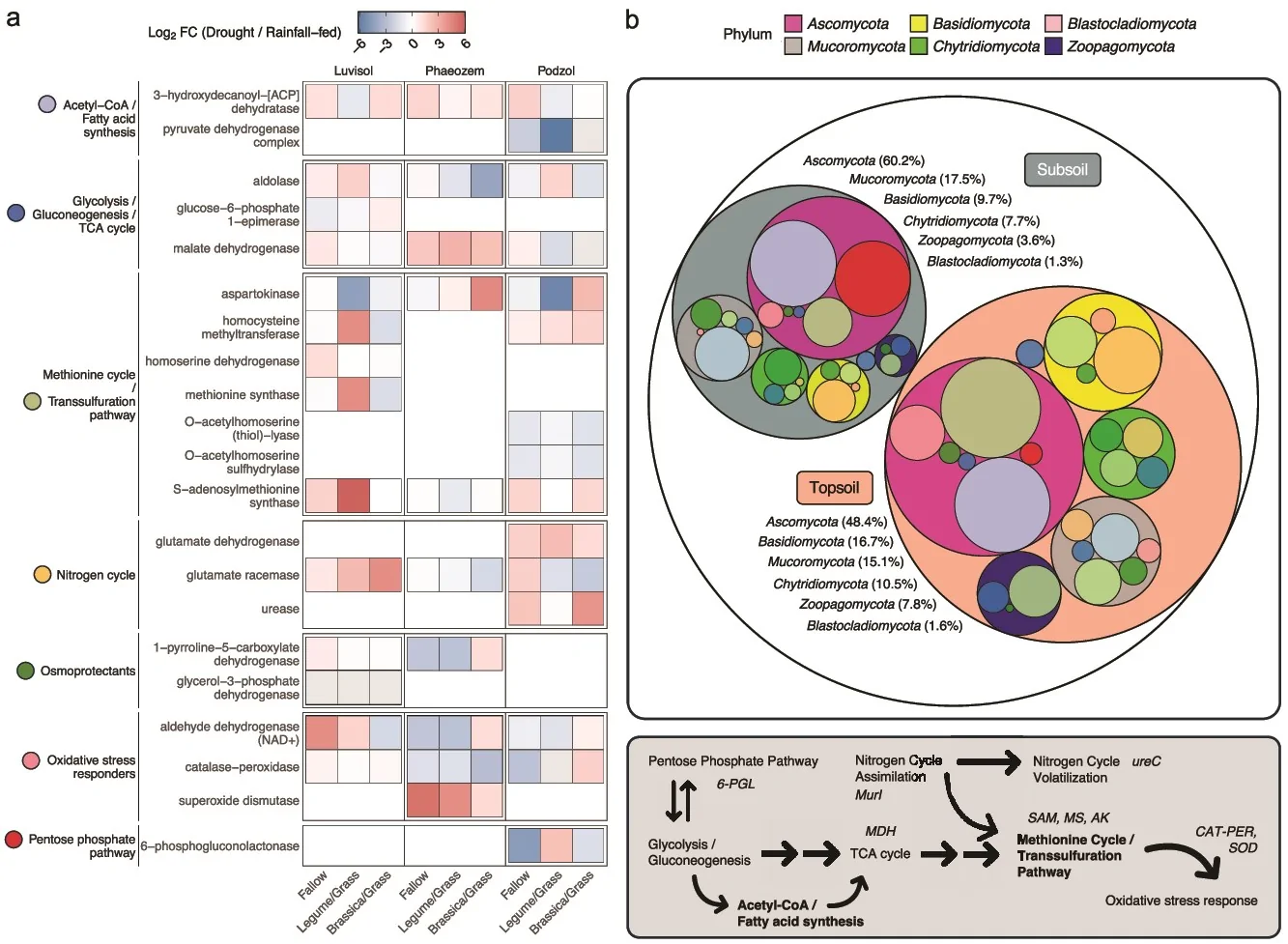
Overview of the functional pathways affected by drought in three soils after cover crop root-channel-reuse across the soil sampling depths. The heatmap is showing the fold change of expressions based on measured protein LFQ intensities for each protein identified under drought and rainfall-fed conditions. Positive logarithmic values of the relative change are denoted by red (higher in drought than in rainfall-fed conditions) and negative values by blue (lower in drought than in rainfall-fed conditions). The small circles next to the names of the biochemical cycles have unique colours, which also have been used to denote the cycles in the circular packing plot, denoting the fungal protein distribution across the soil sampling depths and the contribution from the fungal communities. The significance of the changes in protein expression under drought conditions at each site was calculated using a four-way ANOVA model, significant changes in expression are marked with an asterisk (^*^P < .05) (Supplemental Table S7a consists of a list of proteins which represented significant changes across the parameters of study); At the end, a schematic representation of the dynamics of the functional pathways observed in this study has been portrayed; Legume/Grass: *Fabaceae*/*Poaceae*, Brassica/Grass: *Brassicaceae*/*Poaceae*.

Upon assigning identified proteins to fungal trophic modes (pathotroph, saprotroph, symbiotroph) using FUNGuild, proteins recognized as virulence determinants in pathogenic fungi, such as oxidative stress responders (SOD, CAT-PER, ALDH), membrane transporters (including ABC transporters) and cell-wall-active enzymes (chitinase, glucanases) were expressed at comparable levels across all three guilds rather than being confined to pathotrophs (Table S7c).

### Fungi-mediated lignocellulose breakdown under drought

Lignocellulose is degraded by lignocellulolytic enzymes, primarily hydrolases and some oxidoreductases – classified under different groups of CAZymes. Fungal cellulases play a central role in the decomposition of lignocellulose and complex organic matter, thereby improving resource availability. Metaproteomics analysis revealed that glycoside hydrolases (GHs) were the most abundantly expressed CAZymes and were major contributors to lignocellulose decomposition. They were predominantly derived from the *Ascomycota* classes *Eurotiomycetes* and *Sordariomycetes,* while the other fungal taxa contributed sparsely (Fig. 6a, b).

**Figure 6:**
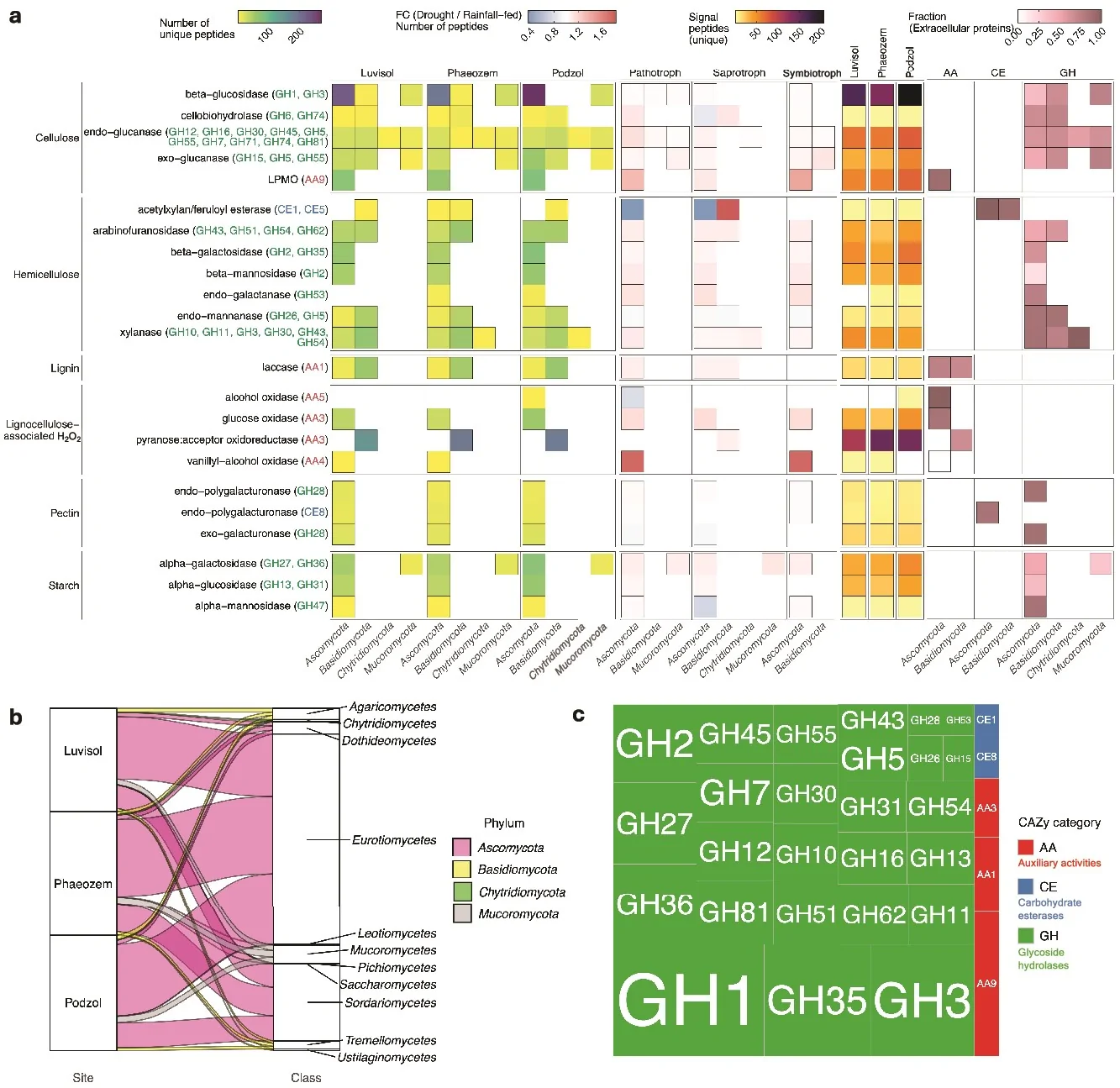
Graphs showing the behaviour of fungal CAZymes under drought stress. (a) The heatmap represents a combination of several heatmaps expressing in the following order: the total number of peptides mapped to fungal CAZymes that act on lignocelluloses or are involved in supportive activities related to lignocellulose degradation; positive fold-change values of the number of peptides from CAZymes under drought against rainfall-fed are denoted by red (higher in drought than in rainfall-fed conditions) and negative values by blue (lower in drought than in rainfall-fed conditions). The CAZymes are linked to the fungal phyla, mentioned at the *x*-axis and at the top of the heatmap contains information about fungal trophic modes (pathotroph, saprotroph and symbiotroph); the total number of signal peptides mapped back to the CAZymes using Phobius; the quantification of the fraction of the number of extracellular proteins, detected by the presence of signal peptides. Further details about the fungal classes linked to these enzymes are represented in the Sankey diagram (b), which we link to the soil types featuring the communities; (c) A treemap representation of the CAZy database-classified categories that had the highest representation in the heatmap. The three colours are for representing the three categories of CAZymes identified—enzymes with AA in red, carbohydrate esterase (CE) in blue, and GH in green. A summary of identified CAZymes is provided in Table S8a–c and the entire list of signal peptides predicted by Phobius are provided in Table S11a–c; Legume/Grass: *Fabaceae*/*Poaceae*, Brassica/Grass: *Brassicaceae*/*Poaceae*.

Enzymes were further categorized according to the lignocellulosic substrates they target. Among the CAZymes, GHs were the most highly expressed, with β-glucosidases (GH1, GH3), xylanases (GH3, GH10, GH11), arabinofuranosidases (GH43, GH51), and α- and β-galactosidase (GH2, GH35) being particularly abundant. These enzymes were largely contributed by *Ascomycota* (Fig. 6a–c, Fig. S8, Table S8a, b). Auxiliary activity (AA) enzymes were the next most expressed group, notably pyranose:acceptor oxidoreductase (AA3) derived from *Basidiomycota* and especially abundant in the Podzol. Most identified CAZymes were extracellular, particularly those affiliated with *Basidiomycota* and *Chytridiomycota* (Fig. 6a, Table S8c). Although *Ascomycota* were the dominant producers of CAZymes, a proportion of their enzymes were predicted to be intracellular (e.g. AA4, GH2). Under drought conditions, pathotrophic and symbiotrophic *Ascomycota* showed increased expression of GH12 and GH74 endoglucanases (Table S8a). In contrast, GH6 cellobiohydrolases were overexpressed by saprotrophic *Basidiomycota* but displayed reduced expression in saprotrophic *Ascomycota*. The auxiliary enzyme lytic-polysaccharide monooxygenase (LPMO; AA9), produced by *Ascomycota* across all trophic modes, was relatively upregulated under drought. Hemicellulose-degrading enzymes, including acetylxylan and feruloyl esterase (CE1), declined under drought in saprotrophic *Ascomycota*, but were overexpressed by saprotrophic *Basidiomycota*. Oxidative stress conditions were associated with increased expression of intracellular vanillyl-alcohol oxidase (AA4), which is involved in vanillin and H_2_O_2_ production. Contrastingly, enzymes involved in lignin and pectin degradation were not significantly affected by drought relative to rainfall-fed. Overall, contributions from *Chytridiomycota* and *Mucoromycota* were substantially lower than those from the *Dikarya* phyla.

## Discussion

This study investigated drought effects on fungal communities and their biochemical pathways within reused winter cover crop root channels subsequently utilized by maize roots. Reusing these root channels facilitate deeper maize root penetration by reducing mechanical resistance from soil particles and improving access to subsoil nutrients. While previous studies demonstrated bacterial enrichment in these systems [5, 8], our findings extend this understanding to fungi, revealing coordinated structural and functional responses shaped by cover crop identity, soil type and moisture availability.

Fungal diversity and abundances varied across soil types, and were strongly influenced by root channel reuse. In the drought-prone Luvisol and Podzol, richness and ITS2 gene copy numbers increased under drought, particularly within *Brassicaceae/Poaceae* root channels, whereas responses differed under fallow and *Fabaceae*/*Poaceae* treatments. Declines in *Chytridiomycota* and *Mucoromycota* under drought are consistent with previous bulk soil observations [49, 50]. In contrast, enrichment of arbuscular mycorrhizal fungi (*Glomeromycetes* and *Mucoromycetes* within *Mucoromycota*) under drought likely reflects their stress-tolerant traits and ability to sustain nutrient exchange with host plants under water limitation [51, 52]. *Zoopagomycota* increased under drought [53], despite having phylogenetic similarity to *Mucoromycota* [54], indicating niche differentiation among zygosporangia-forming lineages. The dominant *Dikarya* (*Ascomycota* and *Basidiomycota*) exhibit limited overall shifts, consistent with their ability to survive under resource limitations through stress-adaptive measures such as sporulation [55, 56]. Associations with soil physicochemical properties support niche partitioning across soil profiles [57]. Fast-growing taxa (*Ascomycota*, *Chytridiomycota,* and *Zoopagomycota*) preferentially exploit labile organic C and available N [58], explaining their positive correlations with TOC and TN in the nutrient-rich Phaeozem and their predominance in the topsoil. Conversely, negative correlations for *Basidiomycota* and *Mucoromycota* with TOC and TN are consistent with adaptation to lower nutrient conditions in subsoils. *Basidiomycota*, known to establish symbiotic relationships with maize roots [59], may benefit from enhanced root penetration into deeper subsoil via reused root channels. These patterns reflect pronounced vertical stratification, whereby topsoil-dominated phyla respond to nutrient availability, while subsoil-dominating communities exhibit adaptations linked to nutrient limitations and symbiotic relationships. The increasing fungal-to-bacterial ratio with depth under drought and in the nutrient-poor Podzol subsoil is consistent with fungi persisting better than bacteria under low-nutrient, water-limited conditions (Fig. S9a). Rare phyla in this study, *Aphelidiomycota* and *Cryptomycota,* prefer low moisture and low pH conditions [60], fitting the negative moisture and pH correlations found here. Drought-associated shifts in trophic modes, characterized by increased representation of saprotrophic and symbiotrophic fungi and reduced pathotrophs, likely reflect improved C sequestration and plant defence responses, potentially supported by the release of plant root-derived metabolites [19, 61].

To elucidate the functional dynamics underlying these structural changes within fungal communities in reused root channels, we used metaproteomics. Upregulation of the fatty acid biosynthesis enzyme 3-hydroxydecanoyl-[ACP]-dehydratase enables membrane restructuring and adjustments in energy storage under drought [18], even in soils such as the Luvisol and Phaeozem that retain substantial moisture. Within *Fabaceae*/*Poaceae* root channels in the drought-affected Podzol, downregulation of *Ascomycota* and *Mucoromycota*-derived PDC enzymes reduces acetyl-CoA flux into the citric acid cycle, suggesting altered energy metabolism under stress [62]. Pyruvate may instead be redirected towards proline biosynthesis, an established osmoprotectant pathway [63]. Downregulation of the proline-degrading enzyme 1-pyrroline-5-carboxylate dehydrogenase (P5CDH) from *Ascomycota*, *Basidiomycota*, *Chytridiomycota*, and *Mucoromycota* in *Fabaceae*/*Poaceae* root channels and fallow favour proline accumulation, enhancing osmotic protection and ROS scavenging [64]. In contrast, P5CDH upregulation in *Brassicaceae*/*Poaceae* root channels may reflect stress tolerance or regulation of proline-induced cell toxicity [65]. Oxidative stress regulation emerged as a prominent feature of fungal drought adaptation. Increased expression of SOD, CAT-PER and ALDH indicates coordinated detoxification of ROS and reactive aldehydes [66]. Treatment-specific expression patterns suggest greater oxidative resilience within *Brassicaceae*/*Poaceae* root channels and potential “stress memory” under drought [67]. Overexpression of glycolytic aldolase in *Blastocladiomycota*, *Chytridiomycota* and *Zoopagomycota* contributes to cellular repair using root exudates as primary C sources for energy [68]. Higher expression of 6-PGL in the pentose phosphate pathway, particularly in the Podzol, implies enhanced flux through the oxidative pentose phosphate pathway branch and thus greater NADPH availability for glutathione-dependent redox buffering [69]. Collectively, these findings indicate integrated regulation of energy metabolism and redox homeostasis under water limitation. The co-occurrence of oxidative stress detoxification (ALDH, CAT-PER and SOD) and osmoprotectant accumulation (notably proline) within the same drought-affected root channels indicates fungi are equipped to survive under several stressors simultaneously, rather than any single stressor, as described for extremely drought-tolerant fungi [70, 71]. These ROS detoxifiers (SOD, CAT-PER, ALDH), along with membrane transporters and cell-wall-active enzymes are recognized virulence determinants in pathogenic fungi [72, 73], yet here they were expressed across pathotrophic, saprotrophic and symbiotrophic trophic modes (Table S7c). This overlap reflects a conserved metabolic response shared between abiotic-stress tolerance and host-associated stress adaptation, rather than any single stressor.

Increased expression of N metabolism enzymes in the Podzol underscores metabolic plasticity in nutrient-poor sandy soils. Upregulation of glutamate dehydrogenase (GDH) and urease suggests alternative N assimilation routes [74], while GDH-mediated deamination may provide C skeletons during limited carbohydrate availability [75, 76]. Concurrent overexpression of methionine cycle and transsulfuration pathway enzymes, such as homocysteine methyltransferase, MeSe and SAM synthase across *Dikarya*, zygosporic *Mucoromycota* and *Zoopagomycota*, and zoosporic *Chytridiomycota* under drought, potentially catalyse SAM to synthesize abiotic stress-regulating polyamines [77] and glutathione, central mediators of abiotic stress tolerance [78]. Fungi dominated SAM synthase and 6-PGL expression in the drought-affected Luvisol and Podzol subsoils, whereas bacterial contributions prevailed in the nutrient-sufficient Phaeozem, indicating a specifically fungal redox contribution where nutrients are scarce (Extended Data, Fig. S9b). Notably, such metabolic adjustments were most pronounced in the coarse-textured Podzol, whereas the silt-rich Phaeozem exhibited comparatively moderate functional restructuring, underscoring the role of soil physical context in mediating drought responses.

Higher CAZyme expression likely reflects increased abundance of *Ascomycota* (*Eurotiomycetes*, *Sordariomycetes*) [79] and *Mucoromycota* (*Mucoromycetes*) [80], all major fungal CAZyme producers (Fig. 2c). The predominance of CAZymes predicted to be secreted (based on signal-peptide detection) suggests an increased potential for mobilisation of complex organic matter and breaking down to simpler forms for emergency metabolic steps. Elevated cellulose-degrading enzymes β-glucosidase, endoglucanase, exoglucanase, and LPMO from *Ascomycota* and *Mucoromycota* potentially enhance breakdown of complex carbohydrates into soluble sugars [81]. The prominence of auxiliary active oxidoreductases, particularly in the Podzol, further suggests plausibly active redox-coupled decomposition under drought, with elevated vanillyl-alcohol oxidase pointing to a possible role in redox balance via regulated H_2_O_2_ production. Hemicellulolytic enzymes such as acetylxylan/feruloyl esterase, arabinofuranosidase and xylanase from *Ascomycota* and *Basidiomycota* probably releases pentoses from hemicellulose, which enters fungal pentose catabolism and the pentose phosphate pathway. They likely support metabolic flexibility under stress, requiring simpler sugars synthesized from degraded hemicelluloses [82]. Complex polysaccharide degradation under drought could therefore be adaptive if the released glucose, xylose and arabinose from CAZyme hydrolysis serve as precursors for osmoprotectant biosynthesis. Their conversion into trehalose and polyols such as mannitol and arabitol is then expected to yield osmoprotectants that lower intracellular water potential, stabilize membranes and proteins, and scavenge ROS during desiccation [83]. Polysaccharide degradation may therefore facilitate osmoregulation alongside C acquisition, although sustained mobilisation of complex organic matter could also deplete soil organic matter stocks, with implications for soil quality.

Fungal responses to drought were functionally convergent despite being phylogenetically dispersed. At the community level, drought responses were not predicted by phylogeny: phylum-level stability could mask divergent class-level shifts, as within the largely unchanged *Dikarya*, where *Laboulbeniomycetes* (*Ascomycota*) and *Malasseziomycetes* (*Basidiomycota*) declined under drought (Fig. 2d). At the functional level, however, such dispersion gave way to convergence, with phylogenetically distinct taxa contributing to interconnected pathways. The central C, methionine/transsulfuration and oxidative stress module (Fig. 5b) not only featured contributions from *Dikarya* but also drew contributions from *Mucoromycota*, *Zoopagomycota*, *Chytridiomycota,* and *Blastocladiomycota*, indicating a convergent functional response to a shared stressor despite their distinct ecological roles.

Beyond this shared protective core, the signatures also describe a continuum between persistence and decomposition of organic C. Downregulated PDC expression, together with proline and other osmoprotectant accumulation in *Fabaceae/Poaceae* root channels, points towards lower labile C flux availability from increased complex polysaccharides as compared to simple polysaccharides, leading to slowing down of PDC expression. By contrast, *Brassicaceae/Poaceae* root channels in the Podzol combined strong oxidative-stress regulation with probable extracellular CAZyme mobilisation (Fig. 6), signalling resource exploitation. Communities therefore combine active resource exploitation with survival measures according to their soil and cover-crop context. These cover crops shaped within-sample diversity and functional contrasts, the highest with *Brassicaceae*/*Poaceae*, but not bulk community composition. Hence, cover crop legacy in reused root channels appear to act by modulating fungal diversity and metabolic activity rather than by taxonomic turnover.

Interpreting microbial dynamics in reused root channels is challenging due to strong spatial and temporal variability in soil moisture and structure, nutrient availability and oxygen content, resulting in context-dependent patterns. Responses also vary with taxonomic resolution. For example, arbuscular mycorrhizal fungi (class *Glomeromycetes*) increased under drought, whereas this trend was not consistent at the broader phylum level (*Mucoromycota*), highlighting ecological differences masked at higher taxonomic groups. A further consideration is the glyphosate (Roundup) applied uniformly to all plots to terminate cover crops before maize sowing. As a constant pre-maize background, it is not likely to have impacted our comparison [84], although its soil-specific influential tendency means any minor residual effect might differ among soils. CAZyme-based interpretations of carbohydrate degradation were done based on the expression of proteins and requires further validation using approaches of biochemical assays (example, enzyme-kinetics assays) or protein-based stable isotope probing (protein-SIP). Moreover, limited studies on soil fungal communities in rhizosphere microsites constrain interpretation. Future work integrating fine-scale spatial sampling with functional analyses is needed for improved understanding of fungal responses to environmental stress.

In conclusion, drought elicited structural and functional adaptations in fungal communities inhabiting the maize rhizosphere within reused cover crop root channels, and these responses were strongly soil-type dependent. In the moisture-limited Podzol and Luvisol, fungi upregulated oxidative-stress defences and downregulated C-N metabolic pathways to conserve energy. By contrast, the water-retentive Phaeozem buffered the cover-crop-driven change in overall expression. Root channels formed by mixtures of *Brassicaceae* and *Poaceae* supported fungal communities that showed strong oxidative stress regulation and improved resource management, suggesting higher drought tolerance. Such cover crop mixtures may represent suitable choices for future cropping strategies under similar environmental conditions. Overall, our findings provide mechanistic insight into how cover crop selection can shape fungal-mediated soil processes. They support strategic cover cropping as an approach to strengthen agroecosystem resilience under climate-induced drought stress.

## Supplementary Material

Supplementary_files_ycag273

## Data Availability

The metaproteomics datasets generated during the current study are available in the PRIDE data repository with the sample metadata, vide PRIDE dataset identifier PXD062138 (https://www.ebi.ac.uk/pride). The raw sequencing data and the respective metadata generated from this study are available under the NCBI BioProject ID PRJNA1240274, which can be accessed using the link: https://www.ncbi.nlm.nih.gov/bioproject/PRJNA1240274. The raw qPCR data, along with the sample metadata, are available on Zenodo under the DOI: 10.5281/zenodo.18889777.
