# Supplementary material for: Fungal response to drought in the maize rhizosphere after reusing cover crop root channels
Source: ISME Commun. 2026 Sep 25;6(1):ycag273. doi: 10.1093/ismeco/ycag273 (PMC13730051; doi:10.1093/ismeco/ycag273)
Supplement: Supplementary file 1 [file supplementary_files_ycag273.zip › Figure_S3_v2_ycag273.pdf]

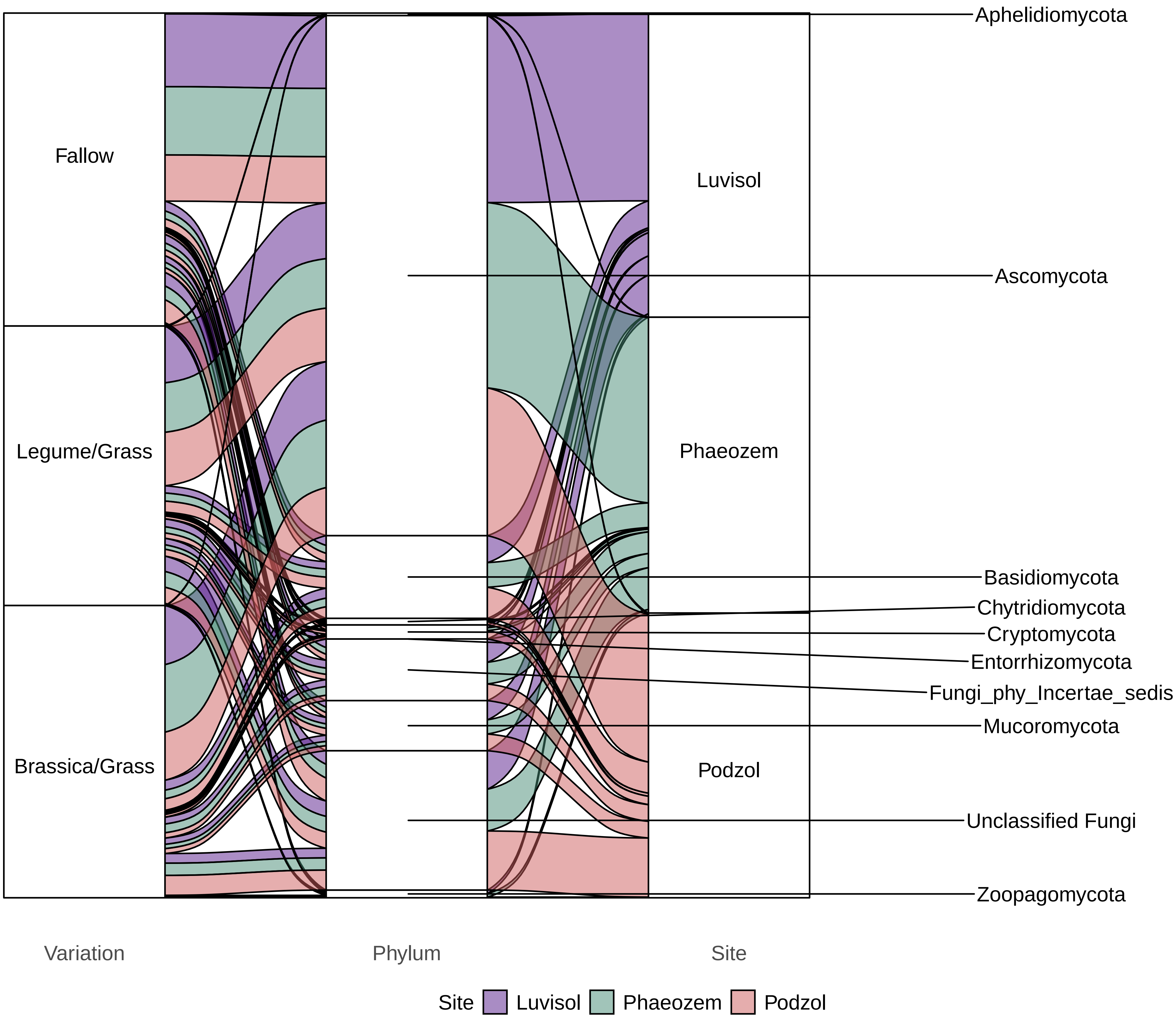
Fallow
Legume/Grass
Brassica/Grass
Luvisol
Phaeozem
Podzol
Aphelidiomycota
Ascomycota
Basidiomycota
Chytridiomycota
Cryptomycota
Entorrhizomycota
Fungi_phy_Incertae_sedis
Mucoromycota
Unclassified Fungi
Zoopagomycota
Variation
Phylum
Site
Site Luvisol Phaeozem Podzol
