# Supplementary material for: Fungal response to drought in the maize rhizosphere after reusing cover crop root channels
Source: ISME Commun. 2026 Sep 25;6(1):ycag273. doi: 10.1093/ismeco/ycag273 (PMC13730051; doi:10.1093/ismeco/ycag273)
Supplement: Supplementary file 1 [file supplementary_files_ycag273.zip › Figure_S7_v2_ycag273.pdf]

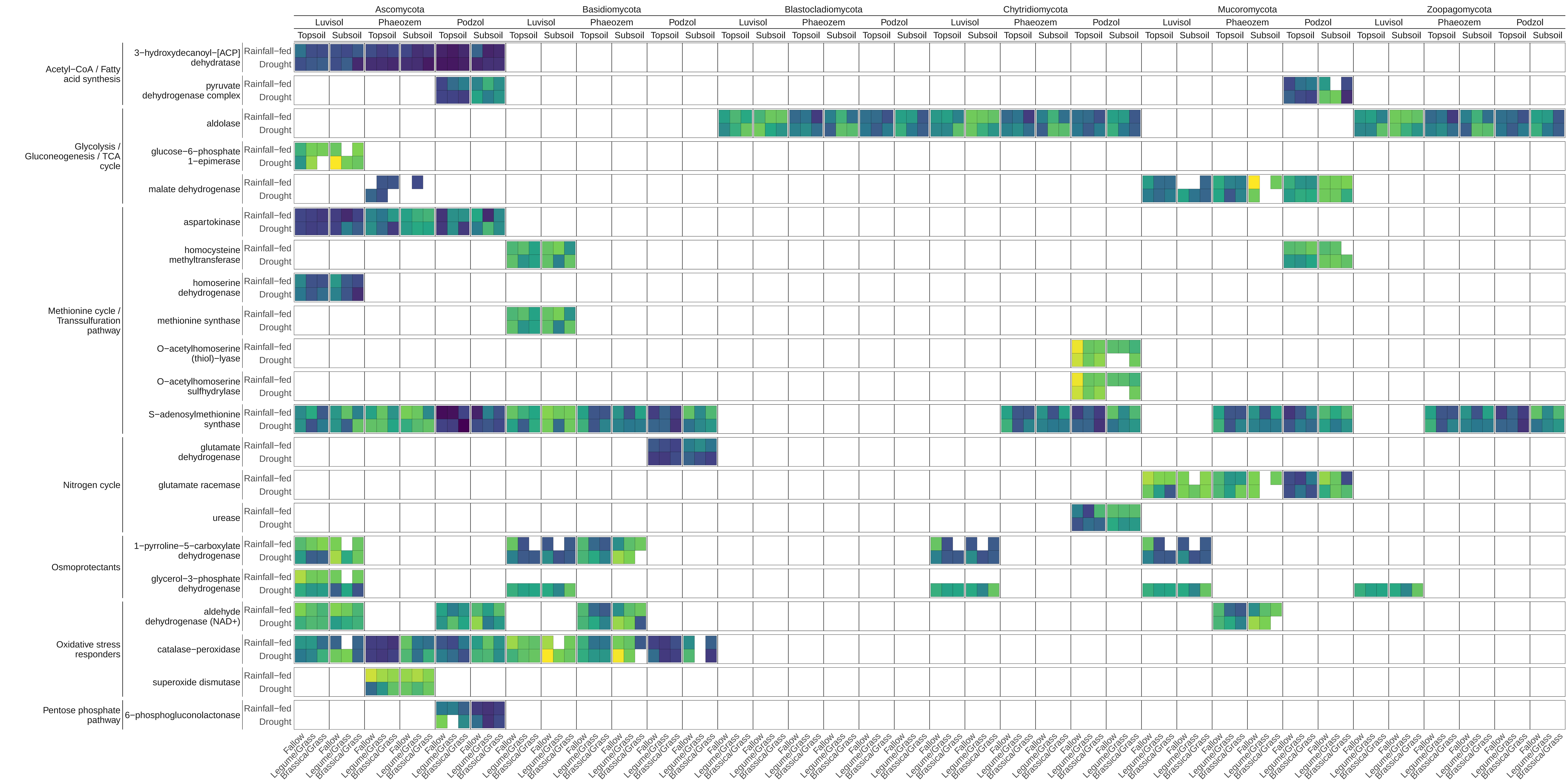

Ascomycota
Basidiomycota
Blastocladiomycota
Chytridiomycota
Mucoromycota
Zoopagomycota
Luvisol
Phaeozem
Podzol
Topsoil
Subsoil
Acetyl−CoA / Fatty acid synthesis
3−hydroxydecanoyl−[ACP] dehydratase
pyruvate dehydrogenase complex
Glycolysis / Gluconeogenesis / TCA cycle
aldolase
glucose−6−phosphate 1−epimerase
malate dehydrogenase
Methionine cycle / Transsulfuration pathway
aspartokinase
homocysteine methyltransferase
homoserine dehydrogenase
methionine synthase
O−acetylhomoserine (thiol)−lyase
O−acetylhomoserine sulfhydrylase
S−adenosylmethionine synthase
Nitrogen cycle
glutamate dehydrogenase
glutamate racemase
urease
Osmoprotectants
1−pyrroline−5−carboxylate dehydrogenase
glycerol−3−phosphate dehydrogenase
Oxidative stress responders
aldehyde dehydrogenase (NAD+)
catalase−peroxidase
superoxide dismutase
Pentose phosphate pathway
6−phosphogluconolactonase
Rainfall−fed
Drought
Fallow
Legume/Grass
Brassica/Grass
