# Supplementary material for: Fungal response to drought in the maize rhizosphere after reusing cover crop root channels
Source: ISME Commun. 2026 Sep 25;6(1):ycag273. doi: 10.1093/ismeco/ycag273 (PMC13730051; doi:10.1093/ismeco/ycag273)
Supplement: Supplementary file 1 [file supplementary_files_ycag273.zip › Supplementary_text_figures_S1-S10_R3_ycag273.docx]

Supplementary information

Fungal response to drought in the maize rhizosphere after reusing cover crop root channels

Ghosh et al.

Correspondence: [nico.jehmlich@ufz.de](mailto:nico.jehmlich@ufz.de), [debjyoti.ghosh@ufz.de](mailto:debjyoti.ghosh@ufz.de)

# Extended Data

# Materials and Methods

### Crop cultivation and sampling regimes

The specific types of rainout shelters covered half of the plot area between the maize rows, reducing 50% rainfall and restricting water infiltration to stemflow. The shelters were installed between the crop rows and below the maize canopy in June, at approximately 50 cm aboveground. This position ensured that the structures did not interfere with leaf-level photosynthesis or direct light interception by the maize plants. They were constructed with a sloped configuration to promote air flow and reduce the risk of heat accumulation or stagnant humidity. The plastic film used was standard greenhouse-grade transparent polyethene, which allows high light transmission while providing effective rain exclusion. A depiction of our rainout shelter setup used in the experiment has been provided within the figure of the experimental plan (Fig. 1).

Before soil sampling, the soil profile was excavated to a depth of 1 m, then excavated 40 cm forward to obtain a fresh profile and fresh maize root system and to prevent any contamination by the neighbouring soil. To compare the difference inflicted by drought on microbial communities in the reused root channels of cover crops by maize, we collected soil samples from a vertical soil profile from the topsoil (0-30 cm) and the subsoil (30-60 cm and 60-90 cm) for two different conditions: 1) maize roots growing in the cover crop root biopores (MCR) from those profiles subjected to drought, and 2) maize roots growing in the MCR in the profiles under rainfall-fed conditions. Maize rhizosphere soil collected along decayed cover crop roots was considered as originating from maize reusing cover crop root channels. The rhizosphere of maize and decayed cover crop roots was defined as extending 2 mm from the root surface. Therefore, the overlapping 2 mm rhizosphere zone of maize and decayed cover crop roots was designated as the sampling area (Fig. S10). The sampling focused on clearly visible maize roots that were closely associated with remaining cover crop root residues, ensuring consistency in sample type across replicates and treatments to reduce subjective variation and avoid systematic error. Additionally, maize rhizosphere samples from the control plot were collected, representing maize roots growing in bulk soil without reusing cover crop root channels.

## Metaproteomics analysis

We searched the MS/MS data against an in-house generated proteome database containing all the defined proteomes in UniProt for the fungi identified by ITS2 gene amplicon sequencing. The database search was performed with Proteome Discoverer™ (v2.5.0.8, Thermo Fisher Scientific) using the SEQUEST-HT algorithm, and all of the outputs are available on PRIDE (EMBL-EBI) [1]. The GhostKOALA and KEGG [2] and COG[3] databases were used for protein functional annotation. Pathways with a minimum of two proteins and a minimum coverage of 5% were selected for downstream processing. Carbohydrate-active enzymes (CAZymes) were identified using Unipept Desktop (v2.0.0, Ghent University) [4]. These identified CAZy enzymes and their preferred substrates provide information regarding rhizo-deposits in the soil profile along cover crop root channels. To estimate the proportion of extracellular carbohydrate-active enzymes (CAZymes), signal peptides were predicted using Phobius [5] and proteins harbouring a signal peptide were classified as extracellular. During database assembly, redundancy was minimised while maintaining taxonomic and functional relevance to avoid repeated assignment of already quantified proteins. Only proteins unambiguously identified by unique peptides and successfully mapped to the UniProt reference database were retained for quantification.

Following pathway categorisation, proteins mapped to biogeochemical cycles of interest helped evaluate shifts associated with soil properties, moisture status, and the re-use of cover crop root biopores, thereby elucidating microbial functional responses.

# Results and Discussion

## Fungal-to-bacterial taxonomic and proteomic trends

In complex environmental systems, there is still a considerable lack of information regarding the comparative presence of fungi and bacteria in the same system in terms of their abundance and contribution to functional pathways. We plotted the fungal-to-bacterial abundance ratio along increasing spatial depth under drought and rainfall-fed conditions in systems with cover crops, calculated as the ratio of ITS2 gene copies per gram of soil to 16S rRNA gene copies per gram of soil measured by qPCR. Bacterial absolute abundances were at least two orders of magnitude higher than fungal abundances, consistent with previous observations [6, 7] (Fig. S9a, Table S12a). Fungal abundances relatively increased under drought as compared to rainfall-fed as depth increased within the root channels of *Brassicaceae/Poaceae* across all soil types, and we presume this may be due to fungal communities inhabiting these root channels being tolerant to oxidative stress. This trend was not observed in the *Fabaceae/Poaceae* root channels, where fungal abundance increased in the Podzol but declined in the Luvisol. The topsoil of the Phaeozem, which is rich in organic matter and likely contains more recalcitrant substrates that fungi decompose more effectively than bacteria [8], explains the significantly higher fungal-to-bacterial ratio compared with the other soil types. The sandy Podzol retains low nutrients, and fungi can survive under low-nutrient conditions better than bacteria [9], which is reflected in the increasing trend of fungal abundance in the Podzol subsoil.

We compared the contribution of fungi and bacteria in reused root channels to biochemical pathways. This was done by quantifying the ratio of LFQ intensities of the identified proteins in our metaproteomics analysis, considering only those proteins detected in both fungi and bacteria. In addition, we only selected proteins associated with the dominant fungal phylum *Ascomycota* and the two dominant bacterial phyla *Actinomycetota* and *Pseudomonadota*. As shown in Fig. S9b, fungal contributions to biochemical pathways increased relatively from the topsoil to the subsoil, possibly due to the ability of fungi to tolerate low-nutrient conditions and therefore function more effectively than bacteria in the subsoil (Table S12b). 6-PGL was contributed relatively more by fungi, particularly *Ascomycota* in the *Fabaceae/Poaceae* root channels, particularly under drought in the Podzol, where it sustains flux through the oxidative branch of the pentose phosphate pathway and therefore support the NADPH generation required for cellular redox homeostasis [10]. The significantly higher expression of SAM synthase from the methionine cycle by fungi (Table S12c) highlights increased contributions under drought versus rainfall-fed conditions in the Luvisol and Podzol, particularly in the subsoils. Together with elevated fungal abundances at these sites, this likely reflects fungal attempts to regulate oxidative stress through glutathione synthesis downstream of the transsulfuration pathway [11–13]. The silt-rich Phaeozem showed relatively higher bacterial contributions to AK and SOD, suggesting enhanced bacterial roles in oxidative stress mitigation in soils with sufficient nutrient availability.

References

1.	Perez-Riverol Y, Bandla C, Kundu DJ, Kamatchinathan S, Bai J, Hewapathirana S, et al. The PRIDE database at 20 years: 2025 update. *Nucleic Acids Research* 2025;**53**:D543–D553. https://doi.org/10.1093/nar/gkae1011

2.	Kanehisa M, Sato Y, Morishima K. BlastKOALA and GhostKOALA: KEGG Tools for Functional Characterization of Genome and Metagenome Sequences. *Journal of Molecular Biology* 2016;**428**:726–731. https://doi.org/10.1016/J.JMB.2015.11.006

3.	Galperin MY, Makarova KS, Wolf YI, Koonin EV. Expanded Microbial genome coverage and improved protein family annotation in the COG database. *Nucleic Acids Research* 2015;**43**:D261–D269. https://doi.org/10.1093/nar/gku1223

4.	Verschaffelt P, Den Bossche T, Martens L, Dawyndt P, Mesuere B. Unipept Desktop: A Faster, More Powerful Metaproteomics Results Analysis Tool. *J Proteome Res* 2021;**20**:2005–2009. https://doi.org/10.1021/acs.jproteome.0c00855.

5.	Kall L, Krogh A, Sonnhammer ELL. Advantages of combined transmembrane topology and signal peptide prediction--the Phobius web server. *Nucleic Acids Research* 2007;**35**:W429–W432. https://doi.org/10.1093/nar/gkm256

6.	Tkacz A, Hortala M, Poole PS. Absolute quantitation of microbiota abundance in environmental samples. *Microbiome* 2018;**6**:110. https://doi.org/10.1186/s40168-018-0491-7

7.	Rousk J, Bååth E, Brookes PC, Lauber CL, Lozupone C, Caporaso JG, et al. Soil bacterial and fungal communities across a pH gradient in an arable soil. *The ISME Journal* 2010;**4**:1340–1351. https://doi.org/10.1038/ismej.2010.58

8.	Wang C, Kuzyakov Y. Mechanisms and implications of bacterial–fungal competition for soil resources. *The ISME Journal* 2024;**18**:wrae073. https://doi.org/10.1093/ismejo/wrae073

9.	Benito‐Carnero G, Gartzia‐Bengoetxea N, Arias‐González A, Rousk J. Low‐quality carbon and lack of nutrients result in a stronger fungal than bacterial home‐field advantage during the decomposition of leaf litter. *Functional Ecology* 2021;**35**:1783–1796. https://doi.org/10.1111/1365-2435.13822

10.	Fuentes-Lemus E, Reyes JS, Figueroa JD, Davies MJ, López-Alarcón C. The enzymes of the oxidative phase of the pentose phosphate pathway as targets of reactive species: consequences for NADPH production. *Biochemical Society Transactions* 2023;**51**:2173–2187. https://doi.org/10.1042/bst20231027

11.	Gong B, Li X, VandenLangenberg KM, Wen D, Sun S, Wei M, et al. Overexpression of S-adenosyl-l-methionine synthetase increased tomato tolerance to alkali stress through polyamine metabolism. *Plant Biotechnology Journal* 2014;**12**:694–708. https://doi.org/10.1111/pbi.12173

12.	Xi C, Pang J, Xue W, Cui Y, Jiang N, Zhi W, et al. Transsulfuration pathway activation attenuates oxidative stress and ferroptosis in sickle primary erythroblasts and transgenic mice. *Communications Biology* 2025;**8**:15. https://doi.org/10.1038/s42003-024-07424-7

13.	Ghosh D, Shi Y, Zimmermann IM, Holzhauser K, von Bergen M, Kaster A-K, et al. Cover Crop Root Channels Promote Bacterial Adaptation to Drought in the Maize Rhizosphere. *Global Change Biology* 2025;**31**:e70512. https://doi.org/10.1111/gcb.70512

# Supplementary figures

Figure S1a: Soil moisture trends in the topsoil (0-30 cm) and subsoil (30-60 cm) along the experimental durations under drought (D) and rainfall-fed (RF) conditions in the Luvisol.

Figure S1b: Soil moisture trends in the topsoil (0-30 cm) and subsoil (30-60 and 60-120 cm) along the experimental durations under D and RF conditions in the Phaeozem.

 Figure S1c: Soil moisture trends in the topsoil (0-30 cm) and subsoil (30-60 and 60-120 cm) along the experimental durations under D and RF conditions in the Podzol.

Figure S2: A bar-chart representing the different values of the physicochemical properties that have been measured at the three soil sampling sites with different soil types – Luvisol (Hohenschulen), Phaeozem (Reinshof) and Podzol (Karkendamm). All values are provided in Table S1.

Figure S3: An alluvial flowchart representing the different variations and the identified fungal phyla in each one of them at the three soil sampling sites. The total number of samples used in the ITS2 gene sequencing analysis were 106 and a metadata have been provided in the Supplementary Table S13.

Figure S4: A heat-tree representation of the differential fungal abundance for the soil types of the three sampling sites. The *log2* values of the relative fungal abundance between site 1 and site 2 are shown by two colours, where ‘purple4’ corresponds to the name of the variation at the top and ‘aquamarine’ to the name on the right side (RStudio colour palettes have been used for colour representation).

Figure S5: A heat-tree representation of the differential fungal abundance in the two layers of soil depth – topsoil and subsoil. The *log2* values of the relative fungal abundance between the layers are shown by two colours, where ‘lightsalmon’ corresponds to topsoil and ‘lightblue4’ to subsoil (RStudio colour palettes have been used for colour representation).

Figure S6: A heat-tree representation of the differential fungal abundance for the cover crop variations and fallow conditions. The *log2* values of the relative fungal abundance between treatments and fallow are shown by two colours, where ‘pink4’ corresponds to the name of the variation at the top and ‘lightgreen’ to the name on the right side (RStudio colour palettes have been used for colour representation).

Figure attached as a vector file (Supplementary Figure S7, PDF)

Figure S7: The extended heatmap from metaproteomics, which includes all the enzymes heavily or sparsely involved in the different functional cycles and fungal phyla corresponding to the enzymes in the three different soil types (Luvisol, Phaeozem and Podzol) under drought and rainfall-fed conditions. For proteomics, *n* = 119. (Additional vector file provided for detailed observations). The significance of the expression of enzymes was measured using MANOVA and have been provided in the Supplementary Table S14.

Figure S8: A summary of the absolute abundance of carbohydrate-active enzymes (CAZymes) represented by label-free quantification (LFQ) intensity, produced by fungal communities categorised by trophic modes defined in the FUNGUILD database under drought and rainfall-fed conditions in our different sampling sites and the different cover crop variations. We have linked the lignocelluloses hydrolysed by these CAZymes on the left-side of the heatmap.

Figure S9: a. A spatial trend of the ratio of ITS2 gene copies in fungi against 16S rRNA gene copies in bacteria across the soil sampling depths (represented by the colour fills of topsoil and subsoil, which are ‘lightsalmon1’ and ‘steelgray’ respectively in the R colour palette) under drought and rainfall-fed conditions for the different cover crop variations (shown by the different coloured trendlines) in the three different soil types; b. A heatmap representing the relative expression of fungal proteins to bacterial proteins for those proteins identified from both organism levels. The colour of the enzyme text is for representing the biochemical cycles to which they contribute. The rows within each facet grid are representing relative expressions under rainfall-fed and drought conditions simultaneously, also indicated by the two small graphical icons to the right of the protein names.

Figure S10: Pictures of the root overlap regions – the root channels of the winter cover crop regions reused by maize for deeper penetration into the soil profile. These were the regions from which the samples were collected for the ‘maize root in cover crop root channels (MCR)’ samples.
